# Characterising the Diffusion Functional Signature of Negative BOLD With Interleaved TMS‐fMRI in the Human Brain

**DOI:** 10.1002/hbm.70629

**Published:** 2026-08-23

**Authors:** Inès de Riedmatten, Arthur P. C. Spencer, Roberto Martuzzi, Vincent Rochas, Jean‐Baptiste Pérot, Filip Szczepankiewicz, Ileana O. Jelescu

**Affiliations:** ^1^ Department of Radiology Lausanne University Hospital (CHUV) Lausanne Switzerland; ^2^ Faculty of Biology and Medicine University of Lausanne (UNIL) Lausanne Switzerland; ^3^ Neuro@Campusbiotech Geneva Switzerland; ^4^ Department of Medical Radiation Physics Lund University Lund Sweden

**Keywords:** apparent diffusion coefficient, diffusion MRI, fMRI, inhibition, motor cortex, negative BOLD, TMS

## Abstract

The coupling between brain excitatory activity and positive blood oxygen level‐dependent (BOLD) responses is well‐established. Although often associated with inhibition, negative BOLD remains partially understood. Moving away from neurovascular coupling, apparent diffusion coefficient (ADC)‐fMRI provides a more direct measure of excitatory activity, possibly mediated by transient cellular deformations. Diffusion‐weighted fMRI (dfMRI), from which ADC‐fMRI derives, combines vascular and microstructural contributions. While decreases in ADC align with positive BOLD, the possible translation of negative BOLD into positive ADC and the ability of ADC‐fMRI to capture inhibitory activity remain unexplored in humans. In this study, we used transcranial magnetic stimulation (TMS)‐fMRI on the right primary motor cortex (M1) to selectively induce contralateral negative BOLD responses, whilst acquiring interleaved fMRI. TMS was applied at 5 Hz, 90% resting motor threshold, for both BOLD‐fMRI and ADC‐fMRI contrasts, in *n* = 12 and *n* = 11 healthy participants, respectively. We replicated previously reported negative BOLD clusters in the contralateral M1 and primary somatosensory cortex (S1). This was accompanied by a negative dfMRI response, but no ADC‐fMRI response, indicating minimal microstructural fluctuations. In ipsilateral M1/S1, no BOLD response was detected while dfMRI revealed a positive cluster, suggesting different sensitivity to the excitatory/inhibitory balance. Overall, combining the findings from BOLD‐fMRI and ADC‐fMRI provides new insights into the vascular and neuronal responses underlying subthreshold TMS and negative BOLD.

## Key Points

- We used interleaved TMS‐fMRI to investigate the diffusion functional signature of negative BOLD.
- In the region with negative BOLD cluster, a negative diffusion‐weighted fMRI cluster, but no corresponding ADC‐fMRI cluster was detected, suggesting minimal microstructural fluctuations.
- The absence of a BOLD cluster at the stimulation site, alongside a strong diffusion‐weighted fMRI cluster (*b* = 200 s mm^−2^) suggests a different sensitivity to the excitatory/inhibitory balance.

## Graphical abstract

We used TMS‐fMRI to study the diffusion signature of negative BOLD. In this region, we observed a negative diffusion‐weighted fMRI cluster but no ADC‐fMRI cluster. Absence of a BOLD cluster at the stimulation site, alongside a positive diffusion‐weighted fMRI cluster (*b* = 200 s mm^−2^), suggests different sensitivity to excitatory‐inhibitory balance.

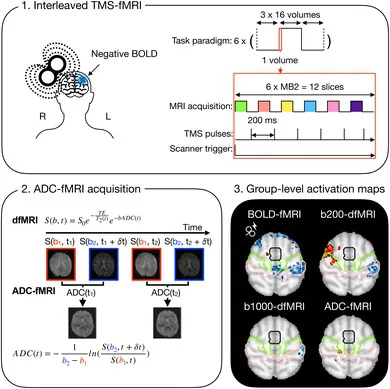

## 1 Introduction

Brain activity is commonly investigated using blood oxygen level‐dependent (BOLD)‐fMRI contrast, which reflects neuronal activity indirectly through neurovascular coupling (Ogawa et al. 1990). In task‐based fMRI, positive BOLD responses are generally interpreted as markers of neural excitation, defined as a transient increase in the probability of firing in a target cell (Epp et al. 2025; Farrant and Kaila 2007). Conversely, negative BOLD responses may reflect neural inhibition, defined as a transient decrease in the probability of firing in a target cell (Farrant and Kaila 2007; Wade 2002; Mullinger et al. 2014).

Recently, apparent diffusion coefficient (ADC)‐fMRI has emerged as a promising functional imaging alternative that links neuronal activity to morphological deformations through so‐called neuromorphological coupling, offering improved spatial and temporal specificity relative to the neurovascular BOLD contrast (Nguyen‐Duc et al. 2025; Spencer et al. 2025). By leveraging sensitivity to water displacement, ADC‐fMRI probes activity‐related microstructural changes. While excitatory neuronal activity is typically associated with a decrease in ADC (Nguyen‐Duc et al. 2025; Spencer et al. 2025; Darquié et al. 2001; Spees et al. 2013; Nunes et al. 2021), likely driven by transient microstructural swelling, the ADC‐fMRI signature corresponding to negative BOLD responses has yet to be characterised.

### 1.1 Blood Oxygen Level‐Dependent fMRI

BOLD‐fMRI is sensitive to changes in the ratio of oxy‐ to deoxyhaemoglobin (Logothetis 2002). During excitatory brain activity, the marked increase in CBF exceeds the concomitant increase in cerebral metabolic rate of oxygen consumption (CMRO_2_). This results in a decrease in the oxygen extraction fraction (OEF) and in deoxyhaemoglobin concentration, captured as an increase in the measured $T_{2}^{\left(*\right)}$‐weighted MRI signal (positive BOLD) (Epp et al. 2025; Fox and Raichle 1986; Buxton et al. 2004).

While positive BOLD is widely acknowledged to result from excitatory activity, the origins of negative BOLD remain unclear. There is substantial evidence that negative BOLD signals are associated with inhibitory neural activity, as shown with local field potential (LFP) and multi‐unit activity (MUA) recordings (Shmuel et al. 2006; Devor et al. 2007). It has been reported that vasoconstriction was correlated to inhibitory synaptic activity, and in some cases, to a reduction in firing rate (Devor et al. 2007). Vasoconstriction would translate into a decrease in CBF with a lesser reduction in CMRO_2_which would in turn result in an increased OEF (Mullinger et al. 2014; Stefanovic et al. 2004). Conversely, it has been suggested that negative BOLD responses may also arise from large increases in neuronal activity (Schridde et al. 2008; Goense et al. 2012), such as during seizures or during the initial dip of the BOLD response. In this case, CMRO_2_ exceeds the increase in CBF, resulting in insufficient oxygen supply. Although less likely, a decrease in CBF due to vascular ‘steal’ by adjacent activated regions is often cited as a potential contributor to negative BOLD (Harel et al. 2002). All of these mechanisms would result in a locally increased deoxyhaemoglobin concentration, a more heterogeneous magnetic field around vessels and thus a decrease in the measured $T_{2}^{\left(*\right)}$‐weighted MRI signal (negative BOLD). Despite the prevalent assumption that negative BOLD reflects inhibitory neuronal activity, the precise neurophysiological mechanisms underlying negative BOLD responses remain a matter of ongoing debate and active investigation (Shmuel et al. 2006).

### 1.2 Apparent Diffusion Coefficient fMRI

ADC timeseries are typically derived from pairs of diffusion‐weighted images acquired with two distinct *b*‐values, which, in combination with other acquisition design features, largely cancels out the T_2_‐weighting and thus BOLD contributions to the ADC time courses (Nguyen‐Duc et al. 2025; Spencer et al. 2025; de Riedmatten, Spencer, and Nguyen‐Duc 2025). The ADC timeseries are calculated according to Equation (1):

(1) $$\mathrm{ADC}\left(t\right)=-\frac{1}{b_{2}-b_{1}}\ln\left(\frac{S\left(b_{2},t+\mathrm{\delta t}\right)}{S\left(b_{1},t\right)}\right)$$

with $S\left(b,t\right)=S_{0}e^{-\frac{\mathrm{TE}}{T_{2}\left(t\right)}}e^{-b\mathrm{ADC}\left(t\right)}$. The two *b*‐values are commonly acquired in an alternating fashion. The corresponding *S*(*b*_1_) and *S*(*b*_2_) timeseries can also be analysed individually and are referred to as diffusion fMRI (dfMRI). By combining T_2_‐ and diffusion‐weighting, the dfMRI time courses retain partial BOLD‐like contrast and sensitivity to microvasculature (via the spin‐echo acquisition (Kim and Ogawa 2012)) in addition to sensitivity to cellular microstructure (via diffusion‐weighting). Diffusion‐weighting also reduces direct contributions from intra‐vascular signals.

While a functional decrease in ADC during excitatory brain activity has been linked to transient neuromorphological alterations, encompassing deformations and swelling in axons, cell bodies, dendrites, synaptic boutons, and astrocytes' processes, as shown in optical imaging studies (Ling et al. 2020; Kwon et al. 2023; Chéreau et al. 2017; Sherpa et al. 2016), the neuromorphological changes associated with negative BOLD have not yet been investigated.

### 1.3 Transcranial Magnetic Stimulation

We used transcranial magnetic stimulation (TMS) combined with fMRI to modulate brain activity while simultaneously acquiring fMRI data, adopting a ‘perturb‐and‐measure’ approach (Siebner et al. 2009). Previous studies have demonstrated that applying unilateral subthreshold repetitive TMS to the primary motor cortex (M1) at 3–5 Hz induces negative BOLD responses in the contralateral M1 (Bestmann et al. 2003, 2004; Shimomura et al. 2018), an effect that has also been implicated in supporting functional recovery in hemiparetic stroke patients (Shimomura et al. 2018). In the context of motor function, this negative BOLD may reflect interhemispheric inhibition, which is thought to enable unilateral movement and high levels of manual dexterity in complex tasks (Stefanovic et al. 2004).

The primary goal of this study is to investigate the ADC‐fMRI response associated with negative BOLD, together with the responses of its constituent diffusion‐weighted fMRI time courses, thereby providing new insights into the microstructural response underlying negative BOLD. As a secondary goal, because negative BOLD response is probably not simply a mirror of positive BOLD response but arises from distinct neurovascular coupling mechanisms (Mullinger et al. 2014), we also aim to use ADC‐fMRI and dfMRI to disentangle neuronal contribution from vascular effects underlying negative BOLD signals.

## 2 Methods

### 2.1 Experiments

This study was approved by the ethics committee of the canton of Vaud, Switzerland (CER‐VD). All participants provided written informed consent. Twelve healthy young adults (age 20–33 years, median 27; 2 females) were scanned. All subjects except one were right handed according to the Edinburgh Handedness Inventory (Oldfield 1971).

**FIGURE 1:**
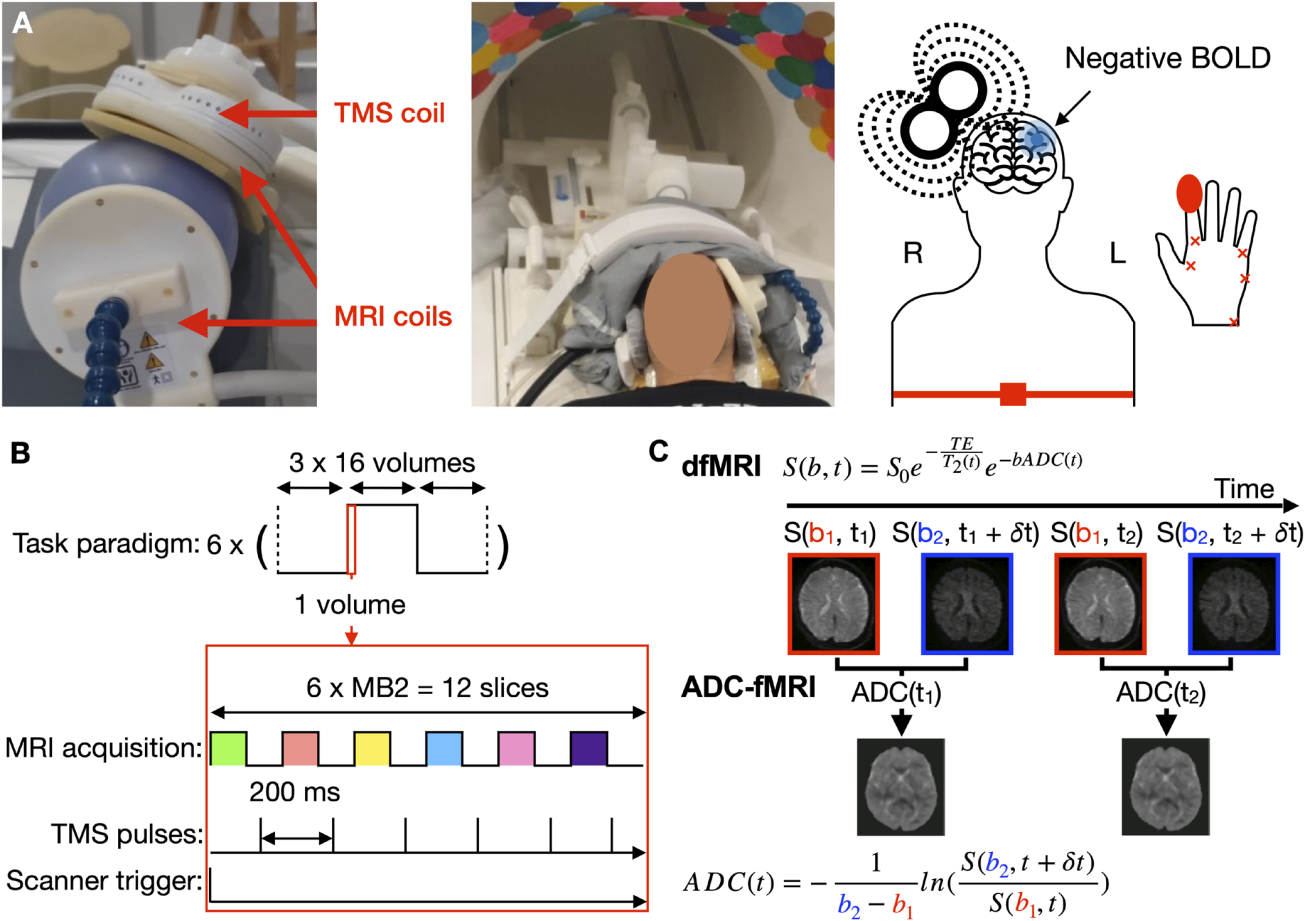
Experimental setup and MRI protocol. (A) TMS‐fMRI setup. A combined TMS coil and seven‐channel MRI coil were positioned over the participant's right primary motor cortex. A second seven‐channel coil was placed symmetrically over the left hemisphere. Surface electromyography (EMG) electrodes were placed on the left hand to localise the motor hotspot and determine the rest motor threshold. Other physiological signals were recorded using a respiration belt and a photoplethysmograph. (B) Interleaved TMS‐fMRI acquisition used for both BOLD‐fMRI and ADC‐fMRI. The task consisted of six epochs of a baseline condition (19.2 s), followed by a TMS block (19.2 s) and a recovery period (19.2 s). During the TMS block, pulses were delivered at 5 Hz between the acquisition of MRI slices to prevent imaging artifacts. One image volume contained 12 slices, acquired with a multiband factor of 2. (C) Diffusion fMRI and ADC‐fMRI acquisition. ADC time courses were derived by combining pairs of diffusion‐weighted volumes acquired at two different *b*‐values *b*_1_ (200 s mm^−2^) and *b*_2_ (1000 s mm^−2^). ADC, apparent diffusion coefficient; BOLD, blood oxygen level‐dependent; dfMRI, diffusion functional MRI.

Prior to the MRI scan, the right M1 hotspot was determined for each subject outside the scanner using single TMS pulses. The scalp location that consistently evoked muscle twitches in the left hand was marked with a cross. Once set up in the MRI scanner, the position of the MR‐compatible TMS coil at the hotspot was confirmed and the resting motor threshold (RMT) was determined as the intensity eliciting identifiable motor‐evoked potentials (MEPs) in half of the occurrences, as recorded from five pre‐gelled electromyography (EMG) electrodes (EL508, BIOPAC Systems Inc., Goleta, US) measuring the interosseus dorsalis primus manus and the abductor digiti minimi muscle groups of the left hand (Figure 1A).

### 2.2 Physiological Monitoring

During the functional scans, photoplethysmography (TSD200‐MRI), respiration belt (TSD201) (BIOPAC Systems Inc., Goleta, US) and EMG data were recorded via a BIOPAC MP160 physiological monitoring system (BIOPAC Systems Inc., Goleta, California, USA). Triggers sent by the MRI scanner at every repetition time (TR) and triggers sent by the transcranial magnetic stimulator at every TR during the TMS blocks were also recorded via the BIOPAC system. AcqKnowledge data acquisition software (BIOPAC Systems Inc) was used to record signals sampled at 10 kHz. The cardiac and respiratory recordings were used to correct for physiological artifacts.

### 2.3 MRI Acquisition

MRI data were acquired using a 3T Siemens Magnetom Prisma with 80 mT/m gradients and 200 T/m/s slew rate. For anatomical reference and tissue segmentation, high‐resolution T_1_‐weighted images were acquired with a 64‐channel head coil, using 3D Magnetization Prepared Rapid Acquisition Gradient Echoes (MPRAGE) with the following parameters: 1 mm^3^ isotropic voxels; 192 × 240 × 256 mm^3^ field of view (FOV); 256 slices; TR 2300 ms; echo time (TE) 2.98 ms; inversion times (TI) 900 ms; flip angle 9°.

A MR‐compatible MRi‐B91 Air Cooled TMS coil (MagVenture A/S, Farum, Denmark) was positioned tangential to the scalp of the subjects on the right M1 for TMS. A 7‐channel (7ch) surface coil compatible with TMS (ALSIX GmbH, Vienna, Austria (Navarro de Lara et al. 2015)) was positioned between the TMS coil and the scalp to receive the MRI signal (Figure 1A). Another 7ch surface coil was positioned symmetrically on the left M1.

For intermediate registration of the fMRI volumes to high‐resolution anatomical T_1_‐weighted images, two anatomical balanced steady state free precession (bSSFP) volumes were acquired: one with the body coil (BC); one with the 7ch coils, with the following parameters: 2 mm^3^ isotropic voxels; 192 × 256 × 256 mm^3^ FOV; 128 slices; TR 5.24 ms; TE 2.33 ms; flip angle 28°.

Two TMS‐fMRI runs (one with ADC‐fMRI and one with BOLD‐fMRI) of 5 min and 48 s each were acquired in each subject. The order of TMS‐fMRI runs was alternated from one subject to the next to avoid any systematic impact of the stimulation in a specific contrast. Image acquisition parameters were matched between the ADC‐ and BOLD‐fMRI runs and were as follows: TR 1200 ms; matrix size: 72 × 72; 12 slices; 3 mm in‐plane resolution; 3 mm slice thickness with 40% slice gap; in‐plane GRAPPA acceleration factor 2; partial Fourier factor 6/8; simultaneous multi‐slice factor 2; resulting in 216 × 216 × 50 mm^3^ partial brain coverage which comprises the M1, supplementary motor area (SMA) and primary sensory cortex (S1).

ADC‐fMRI data were acquired with a custom diffusion‐weighted spin‐echo EPI sequence using spherical b‐tensor encoding with compensation for concomitant gradient effects (Szczepankiewicz, Sjölund, et al. 2019; Szczepankiewicz, Westin, and Nilsson 2019) to enable isotropic functional sensitivity across fibre orientations (Spencer et al. 2025). This diffusion encoding imparts sensitivity to random displacements in all directions of space in a single shot, as opposed to linear encoding which encodes displacements along a single spatial direction at a time. The ADC‐fMRI run consisted of two *b* = 0 volumes, followed by alternating volumes at *b* = 200 and 1000 s mm^−2^ (Figure 1C) at TE = 83 ms. A total of 290 diffusion‐weighted volumes were acquired per run, resulting in 144 ADC timepoints with a temporal resolution of 2.4 s. The individual *b*‐value timeseries (b200‐dfMRI and b1000‐dfMRI) similarly comprised 144 timepoints with the same temporal resolution. BOLD‐fMRI data (Kundu et al. 2017) were acquired with a multi‐echo gradient‐echo EPI sequence (TE 12.60 27.8 43.0 58.2 ms); flip angle 62°. A total of 290 $T_{2}^{*}$‐weighted volumes were acquired per run, with a temporal resolution of 1.2 s.

For both fMRI contrasts, four volumes were acquired with reverse EPI phase encoding for correction of $B_{0}$ field inhomogeneity distortions. Acquisition parameters are summarised in Table S1.

### 2.4 Transcranial Magnetic Stimulation

The TMS paradigm was conducted in a block design comprising six epochs of a baseline condition (19.2 s), followed by a TMS block (19.2 s) and a recovery period (19.2 s) (Figure 1B). During TMS blocks, 5 Hz TMS (biphasic pulse of 280 μs length) was applied on the right M1 at 90% rest motor threshold (RMT), using MagVenture MagPro XP Transcranial Magnetic Stimulator. This paradigm and stimulation frequency were selected to replicate the experimental design introduced by Shimomura et al. (2018) and to ensure sufficient temporal spacing between TMS pulses and the six MRI acquisition blocks of two slices. Additionally, 5 Hz TMS has demonstrated clinical applications across several conditions, including major depressive disorder (Philip et al. 2015; George et al. 2000; Su et al. 2005), post‐traumatic stress disorder (Philip et al. 2016; Carpenter et al. 2018), Parkinson's disease (Siebner et al. 1999, 2000), chronic neuropathic pain (Hirayama et al. 2006; Saitoh et al. 2007), and stroke recovery (Emara et al. 2010). The TMS‐induced current direction was anterior medial (Chen et al. 2003). During stimulation blocks, TMS pulses were interleaved with the MRI acquisition to avoid image artifacts (Figure 1B), as previously reported in Bestmann et al. (2003), Bestmann et al. (2004) and Shimomura et al. (2018). Concretely, after each trigger sent by the MRI scanner at every TR within the stimulation block, an in‐house Matlab script waited 130 ms before sending a train of six pulses at 5 Hz, to allow for the MRI acquisition time (Figure S1). This resulted in pre/post TMS pulse periods of 15/70 and 16/96 ms for ADC‐fMRI and BOLD‐fMRI, respectively. No TMS artifact appeared in the image, confirming that this post‐TMS pulse period was sufficiently long.

### 2.5 Data Analysis

#### 2.5.1 Physiological Recordings

Physiological signals were downsampled to 100 Hz. Spurious peaks or troughs were manually clipped or replaced with the mean value of the signal to remove outliers. Bandpass filtering using Butterworth filter was applied on respiration (4th order, 0.1–1 Hz) and cardiac (7th order, 0.5–3 Hz) data. RETROICOR (Glover et al. 2000) was applied to extract the two first harmonics of respiration and cardiac traces, using physio_calc in AFNI.

#### 2.5.2 MRI Data

T_1_‐weighted and bSSFP images were denoised with spatially adaptive non‐local means filtering with ANTs (Manjón et al. 2010). Then, N4 bias field correction (Tustison et al. 2010) was applied using ANTs, followed by skull‐stripping with Synthstrip (Hoopes et al. 2022).

DfMRI data underwent a preprocessing pipeline depicted in Figure S2, adapted from prior work (Spencer et al. 2025). Denoising was first applied to the magnitude images of the *b* = 200 and *b* = 1000 s mm^−2^ timeseries separately using NORDIC (Moeller et al. 2021). Both g‐factor estimation and PCA‐based denoising were performed with a 7 × 7 × 7 kernel and a step size of 1. Ringing artifacts were then mitigated in all volumes using Gibbs unringing as implemented in MRtrix3 (Kellner et al. 2016; Tournier et al. 2019). Bias field inhomogeneity was corrected using N4 bias field correction (Tustison et al. 2010), with bias field extracted from the *b* = 0 images. Then, despiking was applied to the *b* = 200 and *b* = 1000 s mm^−2^ timeseries separately to remove outliers, using 3dDespike in AFNI. The median percentage of timepoints labelled as ‘big edits’ was smaller than 3.5%. To correct distortion effects, susceptibility off‐resonance field were estimated from the *b* = 0 images with forward and reversed EPI phase‐encoding directions and applied across all diffusion volumes using FSL Topup (Andersson et al. 2003; Jenkinson et al. 2012). Motion correction was performed separately for each *b*‐value timeseries using ANTs (Tustison et al. 2021), with all volumes registered to the first *b* = 0 image. A brain mask was generated from the distortion‐corrected *b* = 0 images using SynthStrip (Hoopes et al. 2022), manually corrected in freeview from Freesurfer, and applied to exclude non‐brain voxels. The functional data were sequentially registered to bSSFP 7ch, bSSFP BC, T_1_, and 2 mm MNI152 standard space, using ANTs (Figure S2). M1, S1 and SMA from the 2 mm Harvard‐Oxford cortical atlas were registered to the functional subject‐space for region‐of‐interest (ROI) analysis. Finally, corrected *b* = 200 and *b* = 1000 timeseries were used to compute an ADC timeseries according to Equation (1). Subsequent analyses were conducted on these ADC‐fMRI timeseries, as well as on the individual *b*‐value timeseries (b200‐dfMRI and b1000‐dfMRI).

For BOLD‐fMRI data, each echo underwent denoising, Gibbs unringing, bias field correction (bias field extracted from the first echo), and despiking procedures. The median percentage of timepoints labelled as ‘big edits’ was smaller than 2%. Motion parameters were estimated from the first‐echo timeseries, and the resulting transformations were then applied to all echoes, using ANTs. A weighted average of echoes was used to calculate an optimally combined signal, using Tedana (including denoising with TEDPCA and TEDICA) (DuPre et al. 2021). Susceptibility off‐resonance field was calculated from the first echo using FSL Topup, and applied to the optimally combined data to correct distortions. The first‐echo images were used to generate a brain mask, which was manually corrected and subsequently applied to remove non‐brain voxels. M1, S1 and SMA from the 2 mm Harvard‐Oxford atlas were registered to the functional subject‐space for ROI analysis.

For quality control of functional runs, absolute displacement from the first volume and framewise displacement between volumes (Power et al. 2012) were calculated from motion correction parameters for BOLD‐fMRI, b200‐dfMRI and b1000‐dfMRI (Table S2). Two epochs with framewise displacement larger than 0.2 mm were discarded in one subject, in b200‐dfMRI, b1000‐dfMRI, and ADC‐fMRI. B200‐dfMRI, b1000‐dfMRI, and ADC‐fMRI data from one participant were excluded because spatially varying field inhomogeneities obscured the signal. Finally, image signal‐to‐noise ratio (SNR) maps, defined as the average denoised signal divided by the standard deviation of the noise components (residuals), are shown in Figure S3.

### 2.6 Statistical Analysis

The timeseries were averaged across voxels within each hemisphere after temporal filtering (> 0.01 Hz) and regressing out motion parameters and physiological signals from the each voxel's timeseries. Epochs were normalised to half their baseline period (9.6 s preceding task onset) and subsequently averaged across epochs and subjects, for the left and right M1/S1 and the bilateral SMA. The power spectral density (PSD) was computed from the mean timeseries using Welch's method implemented in SciPy (Virtanen et al. 2020).

Subject‐level GLM analyses were performed using FSL FEAT (Woolrich et al. 2001), with temporal filtering (> 0.01 Hz), nuisance regression (six motion parameters and the RETROICOR harmonics), and cluster correction (*z* ≥ 2.3, *p* < 0.05), to detect positive or negative association with the stimulation, modelled as a boxcar function. Similar GLM analyses with a boxcar function convolved with the double‐gamma haemodynamic response function (HRF) can be found in Supporting Information: Materials and Methods. For group analysis, the *z*‐score maps were registered to the standard space. More specifically, the affine transformation matrices from the registration steps to T_1_ space were combined and applied as one transformation to minimise interpolation. The registration to MNI standard space and the resampling to 2 mm isotropic resolution were performed in a second step. Group‐level GLMs (Woolrich et al. 2004) were run with FLAME (FMRIB's local analysis of mixed effects) Stage 1 and 2, and cluster correction (*z* ≥ 2.3, *p* < 0.05). Timeseries averaged across significant voxels were also reported, stratified by hemisphere and response polarity. It is important to clarify that these timeseries are derived from averaging either only the positive or the negative significant voxels from individuals. Therefore, they must be interpreted in the context of the relative number of significant voxels of each polarity. The net effect within a given area is captured by plotting the average response across all significant voxels, regardless of polarity.

For M1/S1, we conducted pairwise comparisons of the percentage of significant voxels using two‐tailed unpaired Mann–Whitney *U* tests. Comparisons were performed between ipsilateral positive versus negative, contralateral positive versus negative, contralateral versus ipsilateral positive, and contralateral versus ipsilateral negative conditions. Multiple comparisons across the four tests and four contrasts were controlled using the Benjamini–Hochberg (BH) (Benjamini and Hochberg 1995) procedure to limit the false discovery rate (FDR). For SMA, only comparisons between response polarities were performed, and multiple comparisons across the four contrasts were controlled using BH FDR.

## 3 Results

### 3.1 ROI‐Average Haemodynamic and Diffusion Responses

**FIGURE 2:**
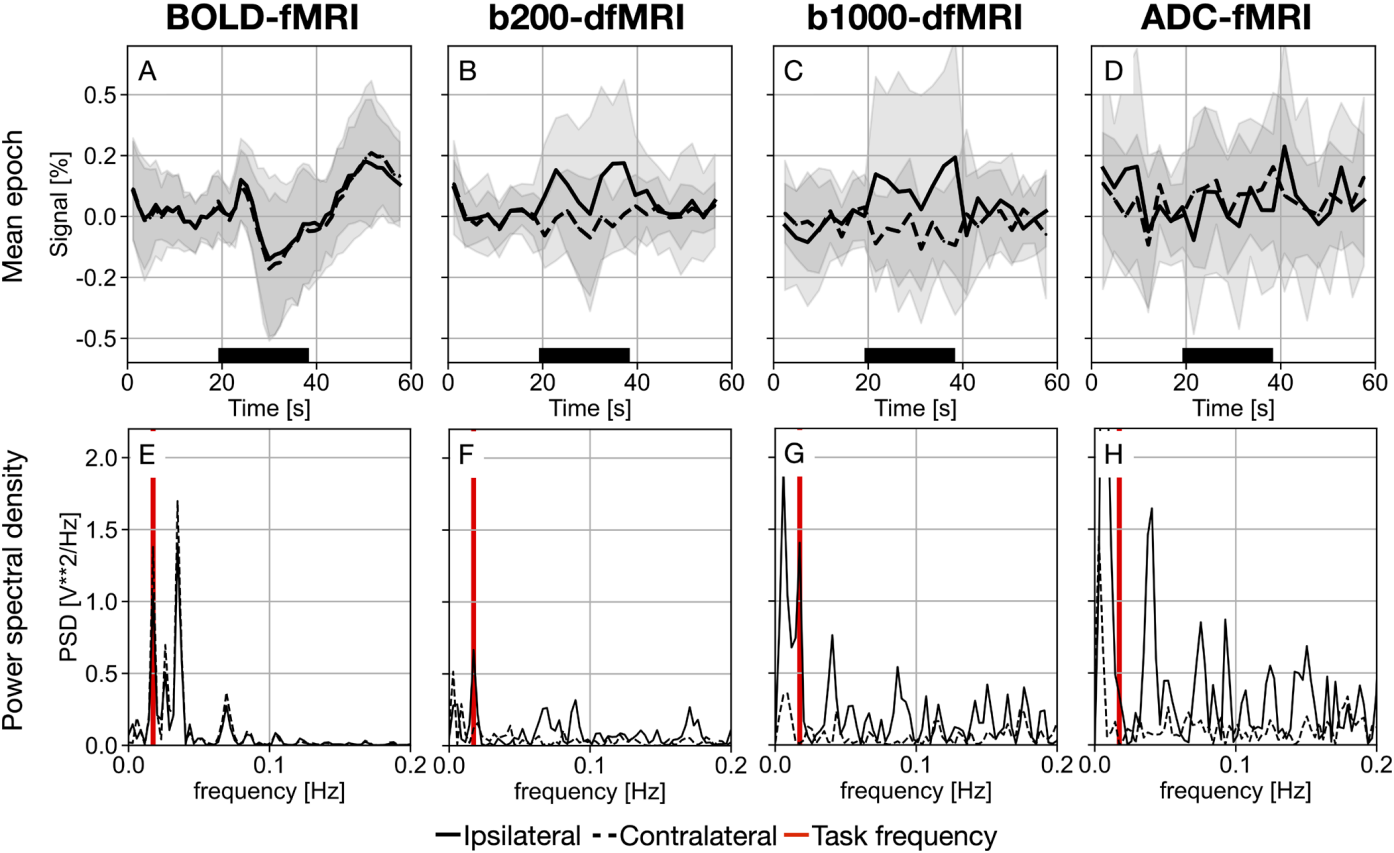
ROI‐average response in M1/S1. Mean epoch time courses (A–D) and corresponding power spectral density (PSD) estimates (E–H) are shown for BOLD‐fMRI (*n* = 12), b200‐dfMRI, b1000‐dfMRI, and ADC‐fMRI (*n* = 11). The timeseries are averaged individually across all primary motor and somatosensory cortex (M1/S1) voxels, and then across subjects. The standard deviation across subjects is depicted as shaded grey areas. TMS duration is indicated by the black bars. The ipsilateral (ipsi, right) hemisphere is shown with solid lines, while the contralateral (contra, left) hemisphere is shown with dashed lines. The task frequency is shown on the PSD as a vertical red line. ADC, apparent diffusion coefficient; BOLD, blood oxygen level‐dependent; dfMRI, diffusion functional MRI.

First, we investigated the ROI‐average response in M1/S1 (Figure 2) and SMA (Figure S4).

For BOLD‐fMRI, the mean M1/S1 signal in both ipsilateral and contralateral hemispheres exhibited a small increase of up to ∼0.1% approximately 5 s after task onset, followed by a decrease below baseline up to ∼−0.2% at 11 s after the task onset (Figure 2A). This biphasic behavior was already observed previously (Sundqvist et al. 2025; Moon et al. 2021). A second positive peak of ∼0.2% appeared around 10 s after task offset. This pattern was reflected in the PSD, which showed a peak at the task frequency and another peak at twice the task frequency (Figure 2E). The SMA mean response showed two positive peaks of ∼0.2% (Figure S4A), at approximately 5 s and 30 s after task onset, with a corresponding PSD peak at twice the task frequency (Figure S4E).

For b200‐ and b1000‐dfMRI timeseries, the M1/S1 response differed between hemispheres (Figure 2B,C). The ipsilateral response increased up to ∼0.2% during the TMS task, with a peak in the PSD at the task frequency (Figure 2F,G), whereas the contralateral response remained essentially flat. In SMA, the response was slightly below baseline (Figure S4B,C).

For ADC‐fMRI, the average M1/S1 response in both hemispheres remained flat (Figure 2D), while the SMA response appeared positive in association with the task (Figure S4D).

### 3.2 Group‐Level Spatial Maps

**FIGURE 3:**
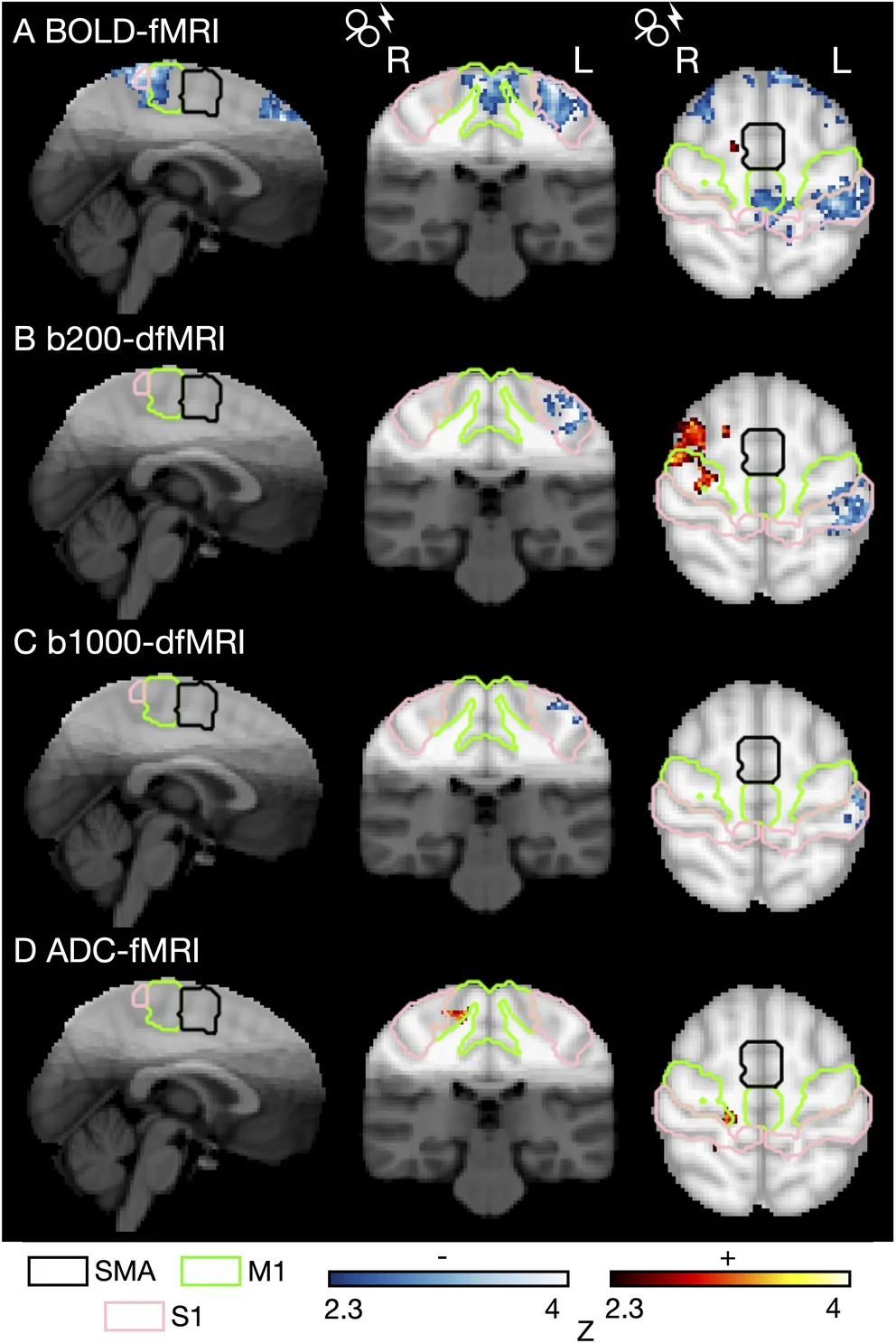
Group‐level activation maps. Activation maps for (A) BOLD‐fMRI (*n* = 12), (B) b200‐dfMRI, (C) b1000‐dfMRI and (D) ADC‐fMRI (*n* = 11). Clusters positively (red) and negatively (blue) correlated with the TMS paradigm are shown (general linear model; cluster‐corrected; *z* ≥ 2.3; *p* < 0.05). The greyscale overlay represents the overlap of partial brain coverage across participants on the 2 mm MNI152 template. Anatomical ROIs from the Harvard–Oxford atlas are outlined: supplementary motor area (SMA, black), precentral gyrus/primary motor cortex (M1, green), and postcentral gyrus/primary somatosensory cortex (S1, pink). ADC, apparent diffusion coefficient; BOLD, blood oxygen level‐dependent; dfMRI, diffusion functional MRI.

We then performed group statistics on the first‐level GLM analyses using a boxcar function to identify significant clusters (Figure 3). All the axial slices are displayed in Figure S5 (boxcar). Positive and negative clusters identified in the group‐level spatial maps are reported in Table 1. Results of GLM analyses using a boxcar function convolved with the double‐gamma HRF are displayed in Figure S5 (HRF).

**TABLE 1:** Significant clusters from group‐statistics on subject‐level GLM analyses. Clusters are shown for BOLD‐fMRI (*n* = 12), b200‐dfMRI, b1000‐dfMRI and ADC‐fMRI (*n* = 11).

Cluster ID	Side	Region	Volume [mm^3^]	MNI coordinate			*p*
				*x*	*y*	*z*	
Positive BOLD‐fMRI							
1	ipsi	Cerebral white matter	6086	16	8	50	3e‐02
Negative BOLD‐fMRI							
3	ipsi	Frontal pole	66,339	18	40	52	4e‐14
3	contra	Frontal pole	66,339	−8	42	54	4e‐14
3	midline	Superior frontal gyrus	66,339	0	46	42	4e‐14
3	ipsi	Middle frontal gyrus	66,339	30	24	54	4e‐14
2	midline	Postcentral gyrus	49,669	0	−38	70	2e‐11
2	midline	Precentral gyrus	49,669	−2	−32	68	2e‐11
2	contra	Postcentral gyrus	49,669	−14	−42	74	2e‐11
1	contra	Postcentral gyrus	38,632	−34	−30	68	1e‐09
1	contra	Precentral gyrus	38,632	−48	−14	42	1e‐09
Positive b200‐dfMRI							
1	ipsi	Precentral gyrus	46,721	42	−6	62	2e‐08
1	ipsi	Middle frontal gyrus	46,721	38	8	56	2e‐08
Negative b200‐dfMRI							
1	contra	Postcentral gyrus	22,831	−48	−28	54	6e‐05
Negative b1000‐dfMRI							
1	contra	Postcentral gyrus	17,388	−40	−28	66	2e‐03
Positive ADC‐fMRI							
1	ipsi	Precentral gyrus	4687	16	−28	62	3e‐02
1	ipsi	Postcentral gyrus	4687	28	−38	64	3e‐02
1	ipsi	Superior parietal lobule	4687	30	−42	64	3e‐02
Cluster corrected, *z* ≥ 2.3, *p* < 0.05							

*Note:* Sides are indicated as ipsilateral (ipsi) and contralateral (contra). Brain regions are defined according to the Harvard–Oxford atlas. Only one or two local maxima per region are shown.

Abbreviations: ADC, apparent diffusion coefficient; BOLD, blood oxygen level‐dependent; dfMRI, diffusion functional MRI.

BOLD‐fMRI yielded negative clusters in M1/S1 at the midline and contralaterally, as well as in both bilateral middle and superior frontal gyri (MFG, SFG) (Figure 3A). Additional negative BOLD clusters were detected in the bilateral frontal pole (FP). In contrast to previous work, no positive (Bestmann et al. 2003, 2004) or negative (Shimomura et al. 2018) clusters were detected in the contralateral dorsal premotor cortex (PMC) or bilateral SMA. No positive BOLD clusters were found in ipsilateral M1/S1, but a small positive cluster was observed in the ipsilateral cerebral white matter near the SMA. HRF modelling detected larger negative clusters in the middle M1/S1 and precuneus, and resulted in more spatially localised frontal clusters within the bilateral FP (Figure S5A, HRF). Conversely, the small positive cluster in cerebral WM revealed by the boxcar response function was not captured when using the HRF.

Both dfMRI contrasts revealed a negative cluster in contralateral M1/S1 (Figure 3B,C), as reported in BOLD‐fMRI. Notably, b200‐dfMRI exhibited an additional positive cluster located between the ipsilateral M1 and MFG, which was absent in b1000‐dfMRI and in BOLD. Similar but smaller clusters were observed with HRF modelling (Figure S5B,C, HRF).

In ADC‐fMRI, a small positive cluster emerged in ipsilateral M1/S1, extending into the superior parietal lobule (SPL), but not overlapping with the b200‐dfMRI positive cluster (Figure 3D). No negative clusters survived correction for multiple comparisons. Overall, clusters detected with b1000‐dfMRI and ADC‐fMRI were relatively small. The mean (±STD) SNR within M1/S1 was 41 ± 11 and 19 ± 5 in b200‐ and b1000‐dfMRI, respectively (Table S3). In comparison, BOLD‐fMRI SNR was 130 ± 32 for TE_1_ and 71 ± 22 for TE_4_. With HRF modelling, no cluster survived correction for multiple comparisons (Figure S5D, HRF).

Examining individual spatial maps revealed substantial inter‐subject variability (Figure S6). The individual BOLD maps obtained using a GLM with a boxcar function convolved with the HRF yielded higher *z*‐scores compared with the boxcar function alone (Figure S7). The opposite effect was observed with diffusion contrasts.

### 3.3 Mean Haemodynamic and Diffusion Responses in Significant Voxels

We also reported the mean responses in subject‐level significant voxels, separated by polarity.

**FIGURE 4:**
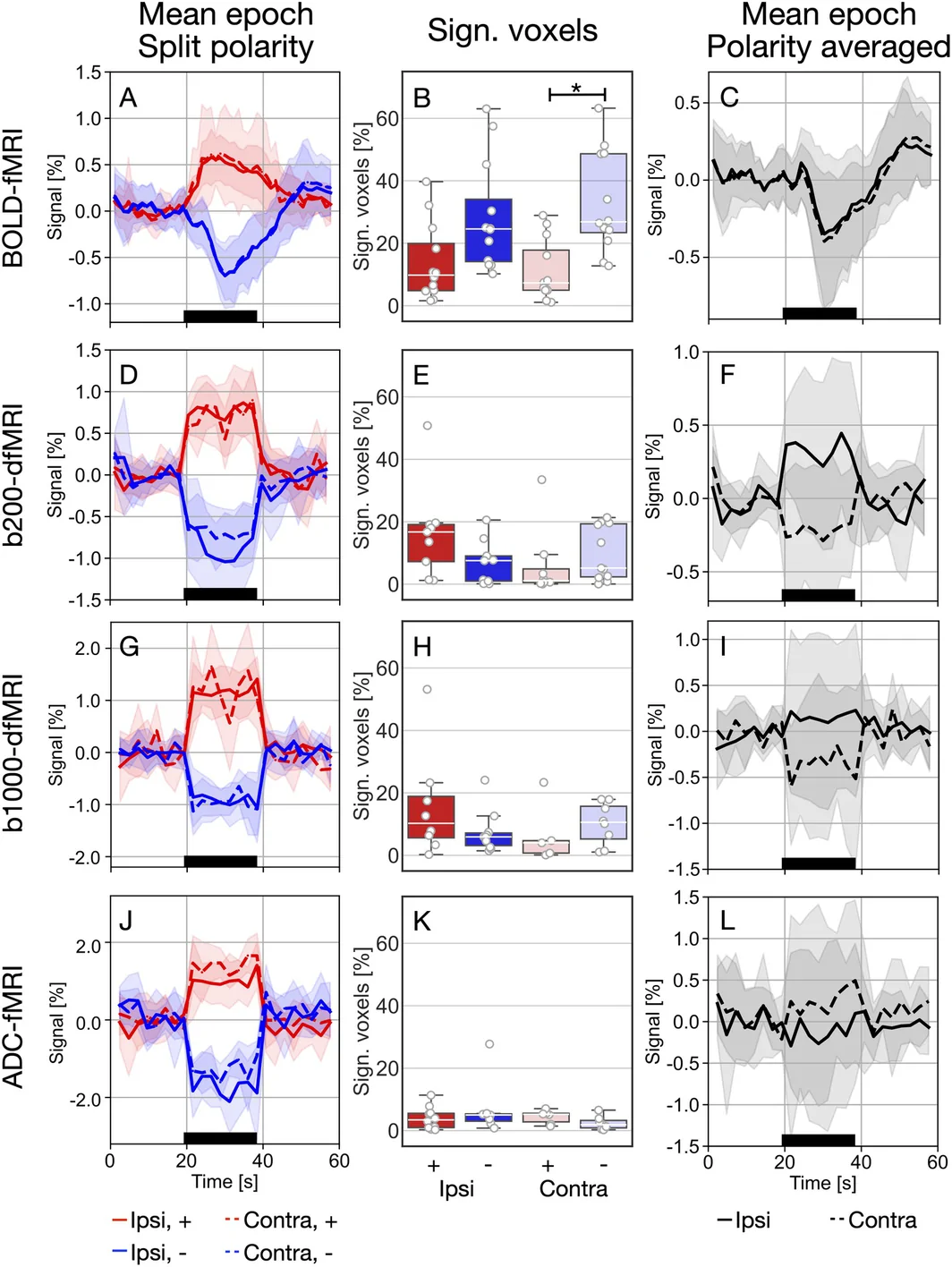
Mean response in significant voxels within M1/S1. Rows correspond to BOLD‐fMRI (*n* = 12), b200‐dfMRI, b1000‐dfMRI, and ADC‐fMRI (*n* = 11). Columns show mean epoch time courses in significant voxels within the primary motor and somatosensory cortex (M1/S1) split by polarity (left), corresponding percentage of significant voxels (middle), and mean epoch time courses averaged across polarities (right). Timeseries were first averaged across significant M1/S1 voxels within each subject and then across subjects. Shaded grey areas indicate the standard deviation across subjects. TMS duration is indicated by black bars. The ipsilateral (ipsi, right) hemisphere is shown with solid lines, and the contralateral (contra, left) hemisphere is shown with dashed lines. Positive (+) and negative (−) associations are depicted in red and blue, respectively. Number of subjects contributing to the averaging: *n*_*ipsi*,+_ = 12, 9, 7, 9, *n*_*ipsi*,−_ = 12, 8, 9, 7, *n*_*control*,+_ = 12, 7, 5, 6 and *n*_*control*,−_ = 12, 9, 7, 6, for BOLD‐fMRI, b200‐dfMRI, b1000‐dfMRI and ADC‐fMRI, respectively. ADC, apparent diffusion coefficient; BOLD, blood oxygen level‐dependent; dfMRI, diffusion functional MRI. Two‐tailed unpaired Mann–Whitney *U* tests, with Benjamini–Hochberg FDR correction for multiple comparisons were performed. *0.01 < *p* ≤ 0.05.

Positive BOLD responses in M1/S1 (Figure 4A) and SMA (Figure S8A) exhibited the characteristic delayed onset and gradual return to baseline, despite using a boxcar response function rather than a haemodynamic response function convolved with a boxcar. Negative BOLD responses displayed an even longer delay to onset and delay to peak (Harel et al. 2002), followed by a positive overshoot approximately 10 s after the task block ended. In SMA, a rebound was observed around 5 s after task onset. When using a boxcar function convolved with the HRF (Figures S9A and S10A), the timeseries displayed a similar shape.

In contrast, dfMRI and ADC‐fMRI average responses (Figure 4D,G,J and Figure S8D,G,J) displayed a sharp onset and return to baseline at the task boundaries, without any notably different shape. In this case, HRF modelling selected different voxels, leading to more gradual signal increases and decreases at the task boundaries (Figures S9D,G,J and S10D,G,J).

Unexpectedly, ipsi‐ and contralateral response amplitudes in M1/S1 closely mirrored each other for all functional contrasts. This is contrary to the expectation that contralateral responses in BOLD and dfMRI would be predominantly negative and would peak at a higher amplitude than hypothesised predominantly positive ipsilateral responses, due to the nature of the subthreshold TMS. The exceptions were the ipsilateral negative responses in b200‐dfMRI and ADC‐fMRI, which were lower than the contralateral response.

However, examining the percentage of significant voxels within M1/S1 (Figure 4, middle) revealed, as expected, a higher proportion of negative BOLD‐fMRI and b1000‐dfMRI responses in contralateral M1/S1. Negative BOLD responses (Figure 4B) were more prevalent than positive responses in both hemispheres, whereas dfMRI showed a trend towards more positive responses ipsilaterally and more negative responses contralaterally (Figure 4E,H), better aligned with expectations from the paradigm design. In the SMA, significant voxels were similarly distributed across positive and negative associations, with a tendency towards more positive BOLD and more negative dfMRI associations (Figure S8, middle). There were significantly more negative than positive significant voxels in contralateral M1/S1 in BOLD‐fMRI (Mann–Whitney *U* test, *U* = 17, *p* = 0.026, FDR‐corrected). All the other tests were non‐significant. When using HRF modelling, the percentage of significant negative BOLD voxels increased, while the percentage of significant positive voxels stayed comparable (Figure S9B). There were significantly more negative than positive significant voxels in contralateral M1/S1 (*U* = 10, *p* = 0.006, FDR‐corrected) and ipsilateral M1/S1 (*U* = 19, *p* = 0.013, FDR‐corrected) in BOLD‐fMRI. For b200‐dfMRI, the percentage decreased in ipsilateral and increased in contralateral, whereas everything decreased in b1000‐dfMRI and in ADC‐fMRI (Figure S9E,H,K). There were significantly more negative significant voxels in ipsilateral versus contralateral M1/S1 in ADC‐fMRI (*U* = 1, *p* = 0.013, FDR‐corrected). All the other tests were non‐significant.

We then disregarded the response polarity and averaged the signal across all significant voxels in M1/S1 (Figure 4, right). In BOLD‐fMRI, the contralateral negative peak was marginally smaller than its ipsilateral counterpart (Δ = −0.05%), whereas the contralateral post‐TMS overshoot was marginally greater than its ipsilateral counterpart (Δ = 0.04%) (Figure 4C). In b200‐dfMRI, there was a clear distinction between the ipsilateral positive responses and the contralateral negative responses (Figure 4F). Similarly, b1000‐dfMRI revealed negative contralateral responses, accompanied by less pronounced ipsilateral positive responses (Figure 4I). For ADC‐fMRI, the contralateral responses were positively associated with the TMS task, whereas the ipsilateral responses were not (Figure 4L). When using HRF instead of boxcar modelling, average significant BOLD‐fMRI responses remained unchanged (Figure S9C). In b200‐dfMRI (Figure S9F), the contralateral positive and negative responses that were captured with HRF modelling cancelled each other and the average significant response was flat, as opposed to the negative contralateral average response found with boxcar modelling. In b1000‐dfMRI, the average significant response captured with HRF modelling was more delayed than the one captured with boxcar modelling (Figure S9F,I).

In SMA (Figure S8, right), the responses were similar to the mean response in the whole ROI (Figure S4), but the negative peaks in b1000‐dfMRI and the positive peak in ADC‐fMRI were more marked (Figure S8I,L). When HRF modelling was applied, the average significant response remained unchanged in BOLD‐fMRI (Figure S10C). Conversely, the amplitudes of the average response were heavily attenuated in b1000‐dfMRI and ADC‐fMRI (Figure S10F,I). In b200‐dfMRI, the significant positive and negative responses captured with boxcar modelling cancelled each other and the average response appeared flat (Figure S8F). When using HRF modelling, the average significant response was negative (Figure S10F).

**FIGURE 5:**
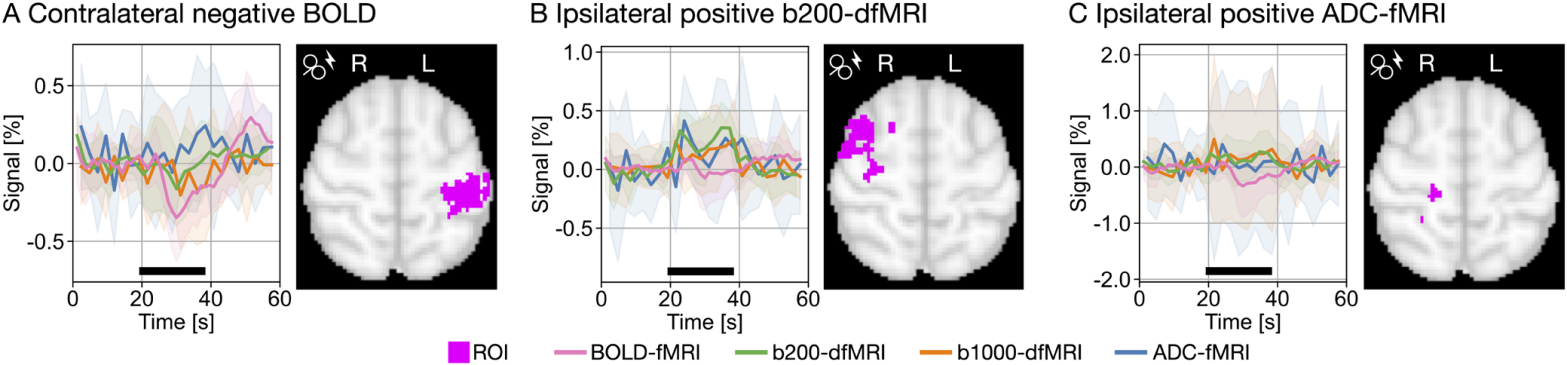
Comparison between contrasts in significant voxels. Responses are shown for BOLD‐fMRI (*n* = 12), b200‐dfMRI, b1000‐dfMRI and ADC‐fMRI (*n* = 11). (A) ROI corresponding to the contralateral negative BOLD‐fMRI cluster shown in M1/S1 in Figure 3A. (B) ROI corresponding to the ipsilateral positive b200‐dfMRI cluster shown in M1/MFG in Figure 3B. (C) ROI corresponding to the ipsilateral positive ADC‐fMRI cluster shown in M1/S1 in Figure 3D. ROIs are shown as pink clusters on the 2 mm MNI152 template. Shaded areas indicate the standard deviation across subjects. ADC, apparent diffusion coefficient; BOLD, blood oxygen level‐dependent; dfMRI, diffusion functional MRI; ROI, region‐of‐interest.

To investigate the average response of all contrasts within clusters identified in Figure 3, masks were defined for the contralateral negative BOLD‐fMRI cluster, the ipsilateral positive b200‐dfMRI cluster, and the ipsilateral positive ADC‐fMRI cluster (Figure 5). Group‐average responses for all contrasts were then computed within each mask. Within the contralateral negative BOLD cluster (Figure 5A), the dfMRI responses showed a slight negative association with the TMS task, while the ADC‐fMRI response showed a positive trend. Within the ipsilateral positive b200‐dfMRI cluster (Figure 5B), the dfMRI and ADC‐fMRI average responses showed a positive association with the task. The BOLD‐fMRI average response showed an initial positive response to the task that rapidly returned to baseline before the task ended. Within the ipsilateral positive ADC‐fMRI cluster (Figure 5C), the dfMRI average responses showed a positive association with the task, while the BOLD‐fMRI average response showed a negative and more delayed trend.

## 4 Discussion

In this study, we applied subthreshold TMS at 5 Hz on the right M1 while simultaneously acquiring fMRI data, following the design of Shimomura et al. (2018). This paradigm has been shown to elicit contralateral negative BOLD in M1/S1. We used this approach to investigate the dfMRI and ADC‐fMRI counterpart of negative BOLD.

We successfully replicated all three BOLD activation clusters reported in Shimomura et al. (2018) that fell within our imaging slab (negative contralateral M1/S1 and bilateral MFG). All other BOLD‐fMRI clusters observed in our study were not previously reported. These include negative clusters in the bilateral FP and in the SFG along the midline, and a positive cluster in cerebral white matter adjacent to the SMA. Despite these differences, the presence of negative clusters distal to the stimulation site confirmed that subthreshold TMS effectively modulated neural circuitry, thereby underscoring the reliability of the experimental setup.

Because BOLD arises from neurovascular coupling, its link to neuronal activity remains indirect. Diffusion fMRI offers complementary insights and may improve our understanding of the mechanisms underlying negative BOLD responses. In particular, ADC‐fMRI is sensitive to excitatory neuronal activity (Nguyen‐Duc et al. 2025; Spencer et al. 2025; Darquié et al. 2001; Spees et al. 2013; Nunes et al. 2021), possibly mediated by concomitant neuromorphological rearrangements (Ling et al. 2020; Kwon et al. 2023; Chéreau et al. 2017; Sherpa et al. 2016), as well as resting‐state activity (de Riedmatten, Spencer, Olszowy, and Jelescu 2025). With appropriate experimental design, ADC‐fMRI is largely independent from vascular contribution (de Riedmatten, Spencer, and Nguyen‐Duc 2025). Conversely, dfMRI time courses combine T_2_‐weighting and diffusion‐weighting, thereby partially reflecting BOLD‐like contrast while reducing direct contributions from intra‐vascular signals (via diffusion‐weighting) and sensitivity to macrovasculature (via spin‐echo acquisition) (Mulkern et al. 2007; Norris 2012). In addition, as opposed to gradient‐echo BOLD, dfMRI spin‐echo acquisition is not sensitive to static dephasing (Mulkern et al. 2007; Norris 2012).

We reported contralateral negative b200‐ and b1000‐dfMRI clusters in M1/S1. The spatial correspondence between BOLD and dfMRI maps contralaterally demonstrates the applicability of the experimental setup beyond conventional BOLD contrast. Similarly, Pérot et al. (2026) reported b200‐dfMRI negative clusters in areas where high frequency visual stimulation induced negative BOLD clusters in rats. They did not find any significant ADC‐fMRI response in these areas. Likewise, in our current study, no contralateral ADC‐fMRI clusters survived statistical thresholding, which may indicate an inherent insensitivity of ADC functional contrast to the mechanisms driving negative BOLD, or insufficient SNR to detect microstructure‐related changes of very small amplitude.

### 4.1 Ipsilateral Effects of Subthreshold TMS‐fMRI

Consistent with previous reports, no ipsilateral M1/S1 positive BOLD cluster was observed during subthreshold TMS (Bestmann et al. 2003, 2004; Shimomura et al. 2018). In contrast, positive BOLD clusters in ipsilateral M1/S1 were previously reported in suprathreshold TMS‐fMRI paradigms (110% RMT, 3–4 Hz, 10 s ON (Bestmann et al. 2003, 2004)). Indeed, TMS generates a phasic electric field in the underlying cortical tissue that depolarises excitable neural elements, most likely myelinated axons (Siebner et al. 2022). When the membrane potential exceeds the threshold membrane potential, it triggers an action potential that, through activation of corticospinal pyramidal neurons, can produce a muscle twitch. Besides affecting the targeted hand motor area, TMS also activates scalp muscles located beneath the coil, which may lead to cutaneous sensation or induce twitch in the scalp or the upper face (Rossi et al. 2009). This neuronal excitatory activity is translated into a positive BOLD response via the haemodynamic response and would be reflected as an ADC decrease and a b200‐dfMRI and b1000‐dfMRI signal increase (Nguyen‐Duc et al. 2025; Spencer et al. 2025; Darquié et al. 2001; Spees et al. 2013; Nunes et al. 2021).

In subthreshold TMS‐fMRI, the absence of ipsilateral positive BOLD clusters is not trivial. Although subthreshold TMS is insufficient to elicit a muscle twitch or a positive BOLD response, it is strong enough to impact remote neuronal activity by affecting the excitatory/inhibitory balance on the contralateral side (Siebner et al. 2022; Di Lazzaro et al. 1998). This apparent paradox has been attributed to (i) the lack of re‐afferent feedback resulting from the absence of TMS‐evoked hand movements, to (ii) haemodynamic responses that remain below background physiological ‘noise’ levels, to (iii) altered haemodynamic responses not detected by conventional analysis methods, or to (iv) mutual cancellation of inhibitory and excitatory effects with no haemodynamic output (Bestmann et al. 2003; Bestmann et al. 2004). Indeed, excitation and inhibition are not temporally distinct, but rather co‐occur within local feedback loops or long‐range feedforward circuits (Isaacson and Scanziani 2011).

Contrary to hypothesis (i), electrophysiological recordings have shown that subthreshold TMS can still induce some motor activity in the hand, even when it is insufficient to produce an overt muscle twitch (Di Lazzaro et al. 1998). It is therefore plausible that, even in the absence of a measurable muscle twitch, some re‐afferent feedback is generated and transmitted back to the brain. However, this signal may be too weak to elicit a measurable BOLD response, which could explain why no significant activation was observed. Supporting the hypotheses (i) and (ii), b200‐dfMRI revealed a robust ipsilateral M1/PMC positive cluster that was not detected with BOLD‐fMRI. This suggests that b200‐dfMRI may be sensitive to subtle task‐locked mechanisms not solely attributable to T_2_ effects, such as morphological changes in neurons, astrocytes or blood vessels. As the recruitment of descending elements (from cortical and motor neurons to muscle fibres), and thus MEP amplitude, scales with stimulus intensity (Kukke et al. 2014; Rossini et al. 2015), an absence of muscle twitch does not mean a complete lack of neural activation or vascular response. Moreover, inhibitory interneurons appear to have a lower firing threshold than pyramidal neurons (Siebner et al. 2022), making firing less energy‐demanding. This could explain the negative BOLD cluster in the contralateral hemisphere in the absence of positive BOLD at the stimulation site. Similarly, scalp sensation elicited underneath the TMS coil could potentially activate somatosensory regions in the ipsilateral hemisphere, and thus explain the observed positive b200‐dfMRI cluster. However, the anatomical location of this cluster is actually more frontal and does not overlap with S1, making this explanation less plausible. The absence of an ipsilateral positive cluster in b1000‐dfMRI is likely attributable to its lower SNR. However, when examining the BOLD‐fMRI average response within the ipsilateral positive b200‐dfMRI cluster, we observed an initial increase of the BOLD amplitude that rapidly returned to baseline before the task ended. This would support the fourth hypothesis, with an early excitatory response that would be cancelled out by a later inhibitory one.

Surprisingly, rather than a negative ADC‐fMRI cluster consistent with the positive b200‐dfMRI cluster in M1/MFG and excitatory activity, we instead detected a positive ADC‐fMRI cluster in ipsilateral M1/S1/SPL. The SPL is known for somatosensory and visuomotor integration, as well as motor learning (Wang et al. 2014). As this cluster is not observed in BOLD, it likely reflects an ADC‐specific signature rather than BOLD contamination. We could speculate that positive ADC represents the inverse of negative ADC and therefore reflects inhibition. Studies of synaptic plasticity and long‐term depression have observed a link between inhibitory stimulation and morphological changes, specifically spine shrinkage, an effect reversible after excitatory stimulation (Zhou et al. 2004). While this evidence derives from long‐term inhibitory processes, short‐term inhibitory processes may also be accompanied by reversible microstructural rearrangements. Such spine shrinkage could contribute to the positive ADC‐fMRI changes observed here, though this remains speculative and untested.

### 4.2 Contralateral Effects of Subthreshold TMS‐fMRI

As demonstrated in previous work (Bestmann et al. 2003, 2004; Shimomura et al. 2018) and replicated in this study, subthreshold TMS applied unilaterally on M1 induces contralateral negative BOLD in M1/S1. Although negative BOLD responses are generally assumed to reflect neuronal inhibition/deactivation (Mullinger et al. 2014; Shmuel et al. 2006; Devor et al. 2007), their underlying mechanisms remain incompletely understood and likely do not represent a simple inverse of positive BOLD (Mullinger et al. 2014; Shmuel et al. 2006).

Shimomura et al. (2018) hypothesised that the observed contralateral negative BOLD cluster resulted from interhemispheric inhibition. Using a paired‐pulse paradigm, Ferbert et al. (1992) showed that EMG activity was reduced when a test pulse on the contralateral M1 followed a conditioning pulse on ipsilateral M1 after a controlled interstimulus interval. Similarly, paired‐pulse transcranial electrical stimulation reduced EMG activity and positive BOLD response in contralateral M1 (Brocke et al. 2008). Such interhemispheric inhibition likely involves excitatory transcallosal projections that excite inhibitory interneurons in contralateral M1 (Siebner et al. 2022; Conti and Manzoni 1994; Takeuchi et al. 2012), or some inhibitory transcallosal projections (Stefanovic et al. 2004; Conti and Manzoni 1994). Following hyperpolarisation of postsynaptic neurons or ‘shunting inhibition’, corticospinal pyramidal neurons in the contralateral M1 exhibit reduced excitability (Newton et al. 2005). In shunting inhibition, the membrane potential may remain unchanged or even become depolarised, yet neuronal excitability is still reduced (Isaacson and Scanziani 2011).

Contralaterally, negative b200‐ and b1000‐dfMRI clusters were observed in M1/S1, spatially overlapping with BOLD‐fMRI negative clusters. This spatial correspondence suggests a shared underlying mechanism governing signal change in this area. In contrast, no positive ADC‐fMRI cluster survived multiple comparison correction in the contralateral M1/S1. Likewise, Pérot et al. (2026) did not report a positive ADC‐fMRI cluster in negative BOLD areas. This absence may reflect limited sensitivity of ADC‐fMRI to the mechanisms underlying negative BOLD, or, alternatively, insufficient statistical power to detect more subtle neuromodulatory effects on microstructure. Should the observed negative BOLD reflect inhibition, a tentative explanation for this preferential ADC‐fMRI sensitivity could be that, during inhibition and even more so during shunting inhibition, the membrane potential does not change as drastically as during action potential firing, and therefore, the associated membrane deformations may also be smaller. However, this interpretation remains speculative and requires direct experimental validation using interferometric imaging.

Regarding the potential lack of statistical power, Spencer et al. (2025) detected ADC‐fMRI responses to a motor task using a comparable sequence and number of subjects, leading to comparable SNR (Table S3). We reiterate that b200‐ and b1000‐dfMRI time courses have combined contributions from diffusion and BOLD‐like (T_2_) effects. Thus, the detection of negative b200‐ and b1000‐dfMRI responses in the contralateral M1/S1 does not automatically translate into an ADC change if the b200‐ and b1000‐dfMRI changes are driven by T_2_ (microvascular) effects rather than neuromorphological effects.

### 4.3 Origin of Contrasts

Notably, ADC‐fMRI and dfMRI time courses exhibited sharp onsets and returns to baseline, suggesting either a diffusion‐weighted contribution rather than simply mirroring the slow haemodynamic response, or alternatively, a preferential sensitivity of spin‐echo acquisition to earlier microvascular changes, as previously observed for positive BOLD (Hulvershorn et al. 2005).

While earlier responses in spin‐echo versus gradient‐echo positive BOLD have been reported, their temporal lead is on the order of ∼1 s (Hulvershorn et al. 2005), which is insufficient to fully explain the larger timing differences observed between the BOLD‐fMRI and the dfMRI/ADC‐fMRI time‐to‐peak, on the order of 2–4 s. No equivalent timing analysis directly comparing spin‐echo and gradient‐echo acquisitions has been reported for negative BOLD responses.

A diffusion contribution to functional contrast could arise from activity‐induced morphological deformations in neurons or astrocytes, but may also originate from vascular microstructure mechanisms, such as vasodilation or vasoconstriction. Regarding vascular microstructure changes, although arterial diameter begins to change within hundreds of milliseconds following neural activation, the time required to reach peak dilation and to return to baseline remains relatively slow, typically occurring several seconds after the task onset or offset (Drew 2019). Furthermore, if fluctuations in vascular microstructure were the source of the dfMRI and ADC‐fMRI contrasts, we would expect a strong association between ADC‐fMRI signals and breath‐hold‐induced brain‐wide vasodilation. However, recent work has demonstrated that this association is weak to non‐existent (de Riedmatten, Spencer, and Nguyen‐Duc 2025).

Using a similar sequence to the one in this study, Spencer et al. (2025) reported sharp onsets in dfMRI time courses, likely reflecting sensitivity (via diffusion‐weighting) to microstructure deformations synchronised with the task. However, they reported delayed offsets in dfMRI timeseries, which may arise from T_2_ contributions through cross‐terms between diffusion‐weighting gradients and susceptibility‐induced background field gradients or vascular microstructure changes. In our study, the sharp nature of both onsets and offsets in dfMRI responses may suggest a contrast mechanism distinct from T_2_.

Overall, sensitivity to non‐vascular microstructure changes appears to be the most plausible explanation for the sharp onsets and offsets observed in the ADC‐fMRI and dfMRI time courses. However, this mechanism alone fails to explain the absence of ipsilateral clusters in b200‐dfMRI and b1000‐dfMRI at the location of the positive ADC‐fMRI cluster.

It should be noted that, when HRF modelling was applied, dfMRI and ADC‐fMRI responses exhibited more gradual onsets and offsets, yielding response profiles more similar to the haemodynamic response, as opposed to the sharp onsets and offsets originally observed using boxcar modelling. However, their onsets remained faster than those observed in BOLD, consistent with the findings of Spencer et al. (2025). Furthermore, fewer significant voxels were detected in dfMRI and ADC‐fMRI when using HRF rather than boxcar modelling, suggesting that these contrasts are more sensitive to task‐locked mechanisms rather than slow T_2_‐related response.

Regarding neuromodulation induced by 5 Hz repetitive TMS, results should be interpreted with caution to avoid overstatement (Hussain and Freedberg 2025). Although a relative consensus suggests that low frequency TMS (< 5 Hz) induces inhibitory effects and high frequency TMS (≥ 5 Hz) induces excitatory effects (Rossini et al. 2015), the underlying mechanisms are considerably more complex (Hussain and Freedberg 2025). The effects of TMS are influenced by stimulation amplitude, frequency, coil orientation, and the total number of pulses delivered, among other factors. For instance, Gorsler et al. (2003) reported a right hand MEP increase after 1800 pulses of TMS (5 Hz, 95% RMT) on the right M1, which would suggest an interhemispheric facilitatory effect. Furthermore, the total number of pulses also appears to be a critical factor. In Peinemann et al. (2004), an increase in right MEP following TMS on the left M1 (5 Hz, 90% RMT) was observed after 1800 pulses, whereas no such effect was detected after 150 pulses. Using a similar methodology, Quartarone et al. (2005) reported that right MEP amplitudes were significantly increased after 900 pulses, but not after 300 or 600 pulses. In this study, participants received 96 TMS pulses per task block, leading to 576 pulses per functional run (six blocks) and 1152 pulses per scanning session (two runs). Taken together, these findings indicate that stimulation frequency and intensity alone are insufficient to account for TMS effects, highlighting the importance of pulse number as an additional determinant. To better characterise the effects of TMS, direct physiological measurements with the same TMS paradigm should be employed, such as MEP amplitude (via paired‐pulse TMS (Ferbert et al. 1992)), deoxyhaemoglobin concentration (with non‐infrared spectroscopy (Mochizuki et al. 2007)), or regional cerebral blood flow (with PET (Fox et al. 1997)).

### 4.4 Limitations

This study presents some limitations and methodological challenges. First, it is limited by its small sample size, which reduces statistical power. As a result, the analyses required more lenient statistical thresholds and should be interpreted with caution. We note however that, at the group‐level analysis, the good anatomical correspondence between significant clusters and expected brain regions suggests the results are not strongly affected by false positives.

Second, delivering precise stimulation, both in terms of target location and intensity, is difficult. While EMG recordings of MEPs following single pulses help validate TMS accuracy, neuronavigation systems would provide even greater precision. Even small deviations from the intended target can lead to an overestimation of the RMT, potentially affecting stimulation efficacy and consistency across participants. Additionally, head displacement relative to the coil can introduce variability in the SNR, potentially obscuring signal variations of interest. This issue can be partially mitigated by motion tracking and real‐time adaptation of TMS intensity (Grosshagauer et al. 2023, 2025). Although no such techniques were employed in the present study, the replication of previously reported results suggests that both the location and intensity of TMS were applied consistently. In addition, ADC‐fMRI and BOLD‐fMRI data were acquired in the same subjects during the same session, enabling direct comparison between the modalities.

Third, due to the geometry of the TMS coils, limiting head motion or vibration after the TMS coil positioning is challenging. During scanning, it is mitigated by fixing the participant's head solidly in place with vacuum pads and an external strap. In GLM analysis, motion parameters and physiological signals were also regressed out to further reduce motion‐related confounds.

Fourth, achieving adequate spatial and temporal resolution while maintaining sufficient SNR within the constraints imposed by the TMS coils remains challenging, although it can be improved through EPI acquisition, multiband acceleration, and partial Fourier. In the present study, the use of partial brain coverage introduced variability due to differences in slab positioning across participants. This partially limits anatomical overlap at the group level, particularly in regions near the edges of the acquired slab. Moreover, the bilateral auditory cortices, typically used as a control region for TMS, are absent from the imaging slab. Interhemispheric inhibition has also been shown to be stronger in the dominant hemisphere (Newton et al. 2005). Although stimulation of the left M1 would therefore be expected to maximise interhemispheric effects in right‐handed participants, technical constraints necessitated stimulation of the right M1 in the present study.

Finally, while TMS is a powerful tool to directly modulate neuronal activity, it represents an artificial modulation which interacts with brain states and does not fully replicate spontaneous engagement of the same network (Hussain and Freedberg 2025; Di Lazzaro et al. 2008).

### 4.5 Conclusions

In conclusion, our subthreshold TMS‐fMRI paradigm broadly replicated previous BOLD findings, including negative BOLD responses in contralateral M1/S1 and at the midline, and absence of positive BOLD responses in ipsilateral M1/S1. We reported for the first time dfMRI and ADC‐fMRI responses to TMS. We found contralateral negative dfMRI clusters that spatially overlapped with the BOLD‐fMRI negative cluster in M1/S1, suggesting a shared underlying mechanism governing this area. However, the sharper onsets and offsets of the diffusion‐weighted fMRI responses may indicate a contribution from mechanisms different from $T_{2}^{*}$ BOLD, in the form of T_2_‐sensitive or diffusion‐sensitive processes. No contralateral ADC clusters were observed, which suggests that the ADC‐fMRI contrast either lacks a negative BOLD counterpart, or is limited by statistical power to capture very weak effects. Ipsilaterally, we reported a positive b200‐dfMRI cluster in M1/PMC, potentially reflecting enhanced sensitivity to subtle ipsilateral task‐locked mechanisms, below the detectability threshold of BOLD, b1000‐dfMRI and ADC‐fMRI contrasts. Overall, dfMRI and ADC‐fMRI offer promising avenues to further disentangle neuronal and vascular contributions to complex responses of subthreshold TMS, though the precise vascular or microstructural mechanisms underlying negative BOLD, negative dfMRI, and positive ADC‐fMRI remain to be fully elucidated.

## Author Contributions

Conceptualisation: I.O.J., I.R. and A.P.C.S. Methodology: I.O.J., I.R., A.P.C.S., R.M., V.R., J.‐B.P. and F.S. Validation: I.R. Formal analysis: I.R. Investigation: I.O.J., I.R., R.M. and V.R. Data curation: I.R. Writing – original draft: I.R. Writing – review and editing: I.R, A.P.C.S., R.M., V.R., J.‐B.P., F.S. and I.O.J. Visualisation: I.R. Supervision: I.O.J. Funding acquisition: I.O.J.

## Funding

Swiss Secretariat for Research and Innovation (SERI), ERC Starting Grant award ‘FIREPATH’ MB22.00032 (I.O.J.), SNSF Eccellenza fellowship no. 194260 (I.O.J.) and Swedish Cancer Society 22 0592 JIA (F.S.).

## Conflicts of Interest

F.S. is co‐inventor in technology related to this research and has financial interests in Random Walk Imaging AB. The other authors declare no conflicts of interest.

## Supporting information


**Table S1:** Acquisition parameters. bSSFP BC = body coil, bSSFP 7ch = 2 × 7 channels MRI receive coils.
**Figure S1:** Data acquisition. The TMS pulses are precisely timed between EPI readouts. The MRI scanner sends a trigger for each acquired volume to a MATLAB script. During task blocks, the script forwards the volume trigger to the TMS generator, which delivers a train of six pulses at 5 Hz. TMS triggers, EMG, pulse oximeter, and respiration belt signals are routed to a Biopac unit and recorded using the ACQknowledge software.
**Figure S2:** Preprocessing pipeline for BOLD‐fMRI and ADC‐fMRI data.
**Figure S3:** Group‐mean image SNR maps for BOLD‐fMRI and ADC‐fMRI data. SNR was calculated as the mean denoised signal divided by the standard deviation of the components removed by denoising (residuals). Group‐mean SNR maps are shown for the four echo times (TE) from BOLD‐fMRI data, and for b = 200 and b = 1000 s mm−2 from the ADC‐fMRI acquisition for comparison. Mean SNR values within the brain are shown in Table S3. Note that image SNR values cannot be calculated for ADC‐fMRI, as this is derived from the constituent b‐value acquisitions.
**Table S2:** Framewise displacement for BOLD‐fMRI and ADC‐fMRI data. The individual framewise displacement (FD) is calculated during the motion correction steps, using ANTS. The mean and max (standard deviation) across subjects of the FD is depicted. Note that the FD cannot be calculated for ADC‐fMRI, as this is derived from the constituent b‐value acquisition.
**Table S3:** Average image SNR for BOLD‐fMRI and ADC‐fMRI data. Mean (standard deviation) across subjects of the average SNR within the M1/S1 regions of the Harvard‐Oxford atlas are shown for the four echo times (TE) from BOLD‐fMRI data, and for b = 200 and b = 1000 s mm−2 from the ADC‐fMRI acquisition for comparison. The last row shows the average SNR within the M1/S1 regions, derived from the motor results in Spencer et al. (2025).
**Figure S4:** ROI‐average response in SMA. Mean epoch time courses (A‐D) and corresponding power spectral density (PSD) estimates (E–H) are shown for BOLD‐fMRI (*n* = 12), b200‐dfMRI, b1000‐dfMRI, and ADC‐fMRI (*n* = 11). The timeseries are averaged individually across all supplementary motor area (SMA) voxels and then across subjects. The standard deviation across subjects is depicted as shaded grey areas. TMS duration is indicated by the black bars. The task frequency is shown on the PSD as a vertical red line. BOLD = blood oxygen level‐dependent, ADC = apparent diffusion coefficient, dfMRI = diffusion functional MRI.
**Figure S5:** Group‐level activation maps (boxcar and HRF models). Activation maps are shown for (A) BOLD‐fMRI (*n* = 12), (B) b200‐dfMRI, (C) b1000‐dfMRI, and (D) ADC‐fMRI (*n* = 11). Clusters positively (red) and negatively (blue) correlated with the TMS paradigm are shown (general linear model; cluster‐corrected; z ≥ 2.3; p < 0.05), displayed on the 2 mm MNI152 template. The task was either modelled with a boxcar function (left column) or a boxcar function convolved with a double‐gamma haemodynamic response function (HRF, right column). Anatomical ROIs from the Harvard‐Oxford atlas are outlined: supplementary motor area (SMA, black), precentral gyrus/primary motor cortex (M1, green), and postcentral gyrus/primary somatosensory cortex (S1, pink). BOLD = blood oxygen level‐dependent, ADC = apparent diffusion coefficient, dfMRI = diffusion functional MRI.
**Figure S6:** Subject‐level activation maps (boxcar model). Activation maps are shown for BOLD‐fMRI, b200‐dfMRI, b1000‐dfMRI and ADC‐fMRI. Clusters showing positive (red) or negative (blue) association with the TMS paradigm are shown (general linear model; cluster‐corrected; z ≥ 2.3; p < 0.05). Anatomical ROIs from the Harvard‐Oxford atlas are outlined: supplementary motor area (SMA, black), precentral gyrus/primary motor cortex (M1, green), and postcentral gyrus/primary somatosensory cortex (S1, pink). BOLD = blood oxygen level‐dependent, ADC = apparent diffusion coefficient, dfMRI = diffusion functional MRI. The dfMRI run of sub‐02 was discarded due to signal dropout.
**Figure S7:** Subject‐level activation maps (HRF model). Activation maps are shown for BOLD‐fMRI, b200‐dfMRI, b1000‐dfMRI and ADC‐fMRI. Clusters showing positive (red) or negative (blue) association with the TMS paradigm are shown (general linear model; cluster‐corrected; z ≥ 2.3; p < 0.05). Anatomical ROIs from the Harvard‐Oxford atlas are outlined: supplementary motor area (SMA, black), precentral gyrus/primary motor cortex (M1, green), and postcentral gyrus/primary somatosensory cortex (S1, pink). BOLD = blood oxygen level‐dependent, ADC = apparent diffusion coefficient, dfMRI = diffusion functional MRI. The dfMRI run of sub‐02 was discarded due to signal dropout.
**Figure S8:** Mean response in significant voxels within SMA (boxcar model). Rows correspond to the BOLD‐fMRI (*n* = 12), b200‐dfMRI, b1000‐dfMRI, and ADC‐fMRI (*n* = 11) contrasts. Columns show mean epoch time courses in significant voxels within the supplementary motor area (SMA) split by polarity (left), corresponding percentage of significant voxels (middle), and mean epoch time courses averaged across polarities (right). Timeseries were first averaged across significant SMA voxels within each subject and then across subjects. Shaded grey areas indicate the standard deviation across subjects. TMS duration is indicated by black bars. Positive (+) and negative (−) associations are depicted in red and blue, respectively. Number of subjects contributing to the averaging: npositive = 12, 8, 5, 5 and nnegative = 12, 7, 7, 3, for BOLD‐fMRI, b200‐dfMRI, b1000‐dfMRI and ADC‐fMRI, respectively. BOLD = blood oxygen level‐dependent, ADC = apparent diffusion coefficient, dfMRI = diffusion functional MRI.
**Figure S9:** Mean response in significant voxels within M1/S1 (HRF model). Rows correspond to the BOLD‐fMRI (*n* = 12), b200‐dfMRI, b1000‐dfMRI, and ADC‐fMRI (*n* = 11) contrasts. Columns show mean epoch time courses in significant voxels within the primary motor and somatosensory cortex (M1/S1) split by polarity (left), corresponding percentage of significant voxels (middle), and mean epoch time courses averaged across polarities (right). Timeseries were first averaged across significant M1/S1 voxels within each subject and then across subjects. Shaded grey areas indicate the standard deviation across subjects. TMS duration is indicated by black bars. The ipsilateral (ipsi, right) hemisphere is shown with solid lines, and the contralateral (contra, left) hemisphere is shown with dashed lines. Positive (+) and negative (−) associations are depicted in red and blue, respectively. Number of subjects contributing to the averaging: nipsi,+ = 12, 9, 8, 8, nipsi,− = 12, 8, 8, 6, ncontra,+ = 12, 6, 3, 7, and ncontra,− = 12, 7, 7, 5, for BOLD‐fMRI, b200‐dfMRI, b1000‐dfMRI and ADC‐fMRI, respectively. BOLD = blood oxygen level‐dependent, ADC = apparent diffusion coefficient, dfMRI = diffusion functional MRI. Two‐tailed unpaired Mann–Whitney U tests, with Benjamini–Hochberg FDR correction for multiple comparisons were performed. *0.01 < p ≤ 0.05; **0.001 < p ≤ 0.01.
**Figure S10:** Mean response in significant voxels within SMA (HRF model). Rows correspond to the BOLD‐fMRI (*n* = 12), b200‐dfMRI, b1000‐dfMRI, and ADC‐fMRI (*n* = 11) contrasts. Columns show mean epoch time courses in significant voxels within the supplementary motor area (SMA) split by polarity (left), corresponding percentage of significant voxels (middle), and mean epoch time courses averaged across polarities (right). Timeseries were first averaged across significant SMA voxels within each subject and then across subjects. Shaded grey areas indicate the standard deviation across subjects. TMS duration is indicated by black bars. Positive (+) and negative (−) associations are depicted in red and blue, respectively. Number of subjects contributing to the averaging: npositive = 12, 6, 4, 3, and nnegative = 12, 6, 4, 3, for BOLD‐fMRI, b200‐dfMRI, b1000‐dfMRI and ADC‐fMRI, respectively. BOLD = blood oxygen level‐dependent, ADC = apparent diffusion coefficient, dfMRI = diffusion functional MRI.

## Data Availability

Raw data are available at https://doi.org/10.5281/zenodo.22011666.

## References

[hbm70629-bib-0001] Andersson, J. L. R. , S. Skare , and J. Ashburner . 2003. “How to Correct Susceptibility Distortions in Spin‐Echo Echo‐Planar Images: Application to Diffusion Tensor Imaging.” NeuroImage 20, no. 2: 870–888. 14568458 10.1016/S1053-8119(03)00336-7

[hbm70629-bib-0089] Benjamini, Y. , and Y. Hochberg . 1995. “Controlling the False Discovery Rate: A Practical and Powerful Approach to Multiple Testing.” Journal of the Royal Statistical Society Series B: Statistical Methodology 57, no. 1: 289–300. 10.1111/j.2517-6161.1995.tb02031.x.

[hbm70629-bib-0002] Bestmann, S. , J. Baudewig , H. R. Siebner , J. C. Rothwell , and J. Frahm . 2003. “Subthreshold High‐Frequency TMS of Human Primary Motor Cortex Modulates Interconnected Frontal Motor Areas as Detected by Interleaved fMRI‐TMS.” NeuroImage 20, no. 3: 1685–1696. 10.1016/j.neuroimage.2003.07.028. 14642478

[hbm70629-bib-0003] Bestmann, S. , J. Baudewig , H. R. Siebner , J. C. Rothwell , and J. Frahm . 2004. “Functional MRI of the Immediate Impact of Transcranial Magnetic Stimulation on Cortical and Subcortical Motor Circuits.” European Journal of Neuroscience 19, no. 7: 1950–1962. 10.1111/j.1460-9568.2004.03277.x. 15078569

[hbm70629-bib-0004] Brocke, J. , S. Schmidt , K. Irlbacher , R. M. Cichy , and S. A. Brandt . 2008. “Transcranial Cortex Stimulation and fMRI: Electrophysiological Correlates of Dual‐Pulse BOLD Signal Modulation.” NeuroImage 40, no. 2: 631–643. 10.1016/j.neuroimage.2007.11.057. 18234515

[hbm70629-bib-0005] Buxton, R. B. , K. Uludağ , D. J. Dubowitz , and T. T. Liu . 2004. “Modeling the Hemodynamic Response to Brain Activation.” NeuroImage 23: S220–S233. 10.1016/j.neuroimage.2004.07.013. 15501093

[hbm70629-bib-0006] Carpenter, L. L. , C. Conelea , A. R. Tyrka , et al. 2018. “5 Hz Repetitive Transcranial Magnetic Stimulation for Posttraumatic Stress Disorder Comorbid With Major Depressive Disorder.” Journal of Affective Disorders 235: 414–420. 10.1016/j.jad.2018.04.009. 29677606 PMC6567988

[hbm70629-bib-0007] Chen, R. , D. Yung , and J.‐Y. Li . 2003. “Organization of Ipsilateral Excitatory and Inhibitory Pathways in the Human Motor Cortex.” Journal of Neurophysiology 89, no. 3: 1256–1264. 10.1152/jn.00950.2002. 12611955

[hbm70629-bib-0008] Chéreau, R. , G. E. Saraceno , J. Angibaud , D. Cattaert , and U. V. Nägerl . 2017. “Superresolution Imaging Reveals Activity‐Dependent Plasticity of Axon Morphology Linked to Changes in Action Potential Conduction Velocity.” Proceedings of the National Academy of Sciences of the United States of America 114, no. 6: 1401–1406. 10.1073/pnas.1607541114. 28115721 PMC5307438

[hbm70629-bib-0009] Conti, F. , and T. Manzoni . 1994. “The Neurotransmitters and Postsynaptic Actions of Callosally Projecting Neurons.” Behavioural Brain Research 64, no. 1: 37–53. 10.1016/0166-4328(94)90117-1. 7840891

[hbm70629-bib-0010] Darquié, A. , J.‐B. Poline , C. Poupon , H. Saint‐Jalmes , and D. le Bihan . 2001. “Transient Decrease in Water Diffusion Observed in Human Occipital Cortex During Visual Stimulation.” Proceedings of the National Academy of Sciences of the United States of America 98, no. 16: 9391–9395. 10.1073/pnas.151125698. 11459931 PMC55431

[hbm70629-bib-0011] de Riedmatten, I. , A. P. C. Spencer , and J. Nguyen‐Duc . 2025. “Evaluating the Dependence of ADC‐fMRI on Haemodynamics in Breathhold and Resting‐State Conditions.” Imaging Neuroscience 3: 1020. 10.1162/IMAG.a.1020. PMC12630321 41278581

[hbm70629-bib-0012] de Riedmatten, I. , A. P. C. Spencer , W. Olszowy , and I. O. Jelescu . 2025. “Apparent Diffusion Coefficient fMRI Shines Light on White Matter Restingstate Connectivity Compared to BOLD.” Communications Biology 8, no. 1: 1–13. 10.1038/s42003-025-07889-0. 40091123 PMC11911413

[hbm70629-bib-0013] Devor, A. , P. Tian , N. Nishimura , et al. 2007. “Suppressed Neuronal Activity and Concurrent Arteriolar Vasoconstriction May Explain Negative Blood Oxygenation Level‐Dependent Signal.” Journal of Neuroscience: The Official Journal of the Society for Neuroscience 27, no. 16: 4452–4459. 10.1523/JNEUROSCI.0134-07.2007. 17442830 PMC2680207

[hbm70629-bib-0014] Di Lazzaro, V. , D. Restuccia , A. Oliviero , et al. 1998. “Magnetic Transcranial Stimulation at Intensities Below Active Motor Threshold Activates Intracortical Inhibitory Circuits.” Experimental Brain Research 119, no. 2: 265–268. 10.1007/s002210050341. 9535577

[hbm70629-bib-0015] Di Lazzaro, V. , U. Ziemann , and R. N. Lemon . 2008. “State of the Art: Physiology of Transcranial Motor Cortex Stimulation.” Brain Stimulation 1, no. 4: 345–362. 10.1016/j.brs.2008.07.004. 20633393

[hbm70629-bib-0016] Drew, P. J. 2019. “Vascular and Neural Basis of the BOLD Signal.” Current Opinion in Neurobiology 58: 61–69. 10.1016/j.conb.2019.06.004. 31336326 PMC6859204

[hbm70629-bib-0017] DuPre, E. , T. Salo , Z. Ahmed , et al. 2021. “TE‐Dependent Analysis of Multi‐Echo fMRI With* Tedana.” Journal of Open Source Software 6, no. 66: 3669.

[hbm70629-bib-0018] Emara, T. H. , R. R. Moustafa , N. M. Elnahas , et al. 2010. “Repetitive Transcranial Magnetic Stimulation at 1 Hz and 5 Hz Produces Sustained Improvement in Motor Function and Disability After Ischaemic Stroke.” European Journal of Neurology 17, no. 9: 1203–1209. 10.1111/j.1468-1331.2010.03000.x. 20402755

[hbm70629-bib-0019] Epp, S. M. , G. Castrillón , B. Yuan , J. Andrews‐Hanna , C. Preibisch , and V. Riedl . 2025. “BOLD Signal Changes Can Oppose Oxygen Metabolism Across the Human Cortex.” Nature Neuroscience 29: 1225–1236. 10.1038/s41593-025-02132-9. 41402521 PMC13156032

[hbm70629-bib-0020] Farrant, M. , and K. Kaila . 2007. “The Cellular, Molecular and Ionic Basis of GABAA Receptor Signalling.” In Gaba and the Basal Ganglia. Progress in Brain Research, edited by J. M. Tepper , E. D. Abercrombie , and J. P. Bolam , vol. 160, 59–87. Elsevier. 10.1016/S0079-6123(06)60005-8. 17499109

[hbm70629-bib-0021] Ferbert, A. , A. Priori , J. C. Rothwell , B. L. Day , J. G. Colebatch , and C. D. Marsden . 1992. “Interhemispheric Inhibition of the Human Motor Cortex.” Journal of Physiology 453, no. 1: 525–546. 10.1113/jphysiol.1992.sp019243. 1464843 PMC1175572

[hbm70629-bib-0023] Fox, P. , R. Ingham , M. S. George , et al. 1997. “Imaging Human Intra‐Cerebral Connectivity by PET During TMS.” Neuroreport 8, no. 12: 2787. 9295118 10.1097/00001756-199708180-00027

[hbm70629-bib-0022] Fox, P. T. , and M. E. Raichle . 1986. “Focal Physiological Uncoupling of Cerebral Blood Flow and Oxidative Metabolism During Somatosensory Stimulation in Human Subjects.” Proceedings of the National Academy of Sciences of the United States of America 83, no. 4: 1140–1144. 10.1073/pnas.83.4.1140. 3485282 PMC323027

[hbm70629-bib-0024] George, M. S. , Z. Nahas , M. Molloy , et al. 2000. “A Controlled Trial of Daily Left Prefrontal Cortex TMS for Treating Depression.” Biological Psychiatry 48, no. 10: 962–970. 10.1016/s0006-3223(00)01048-9. 11082469

[hbm70629-bib-0025] Glover, G. H. , T.‐Q. Li , and D. Ress . 2000. “Image‐Based Method for Retrospective Correction of Physiological Motion Effects in fMRI: RETROICOR.” Magnetic Resonance in Medicine 44, no. 1: 162–167. 10.1002/1522-2594(200007)44:1<162::AID-MRM23>3.0.CO;2-E. 10893535

[hbm70629-bib-0026] Goense, J. , H. Merkle , and N. K. Logothetis . 2012. “High‐Resolution fMRI Reveals Laminar Differences in Neurovascular Coupling Between Positive and Negative BOLD Responses.” Neuron 76, no. 3: 629–639. 10.1016/j.neuron.2012.09.019. 23141073 PMC5234326

[hbm70629-bib-0027] Gorsler, A. , T. Bäumer , C. Weiller , A. Münchau , and J. Liepert . 2003. “Interhemispheric Effects of High and Low Frequency rTMS in Healthy Humans.” Clinical Neurophysiology 114, no. 10: 1800–1807. 10.1016/S1388-2457(03)00157-3. 14499741

[hbm70629-bib-0028] Grosshagauer, S. , M. Woletz , M. Vasileiadi , et al. 2023. “Motion Tracking for Real‐Time Measurements of Coil‐Cortex Distance: A Novel Approach for Improving Dose Consistency in Concurrent TMS/fMRI.” Brain Stimulation: Basic, Translational, and Clinical Research in Neuromodulation 16, no. 1: 323. 10.1016/j.brs.2023.01.600.

[hbm70629-bib-0029] Grosshagauer, S. , M. Woletz , D. Zeman , et al. 2025. “E‐Field‐Guided Intensity Adjustment for Motion Compensation in Concurrent TMS‐fMRI.” Brain Stimulation: Basic, Translational, and Clinical Research in Neuromodulation 18, no. 1: 425. 10.1016/j.brs.2024.12.623.

[hbm70629-bib-0030] Harel, N. , S. P. Lee , T. Nagaoka , D. S. Kim , and S. G. Kim . 2002. “Origin of Negative Blood Oxygenation Level—Dependent fMRI Signals.” Journal of Cerebral Blood Flow & Metabolism 22, no. 8: 908–917. 10.1097/00004647-200208000-00002. 12172376

[hbm70629-bib-0031] Hirayama, A. , Y. Saitoh , H. Kishima , et al. 2006. “Reduction of Intractable Deafferentation Pain by Navigation‐Guided Repetitive Transcranial Magnetic Stimulation of the Primary Motor Cortex.” Pain 122, no. 1: 22. 10.1016/j.pain.2005.12.001. 16495011

[hbm70629-bib-0032] Hoopes, A. , J. S. Mora , A. V. Dalca , B. Fischl , and M. Hoffmann . 2022. “SynthStrip: Skull‐Stripping for Any Brain Image.” NeuroImage 260: 119474. 35842095 10.1016/j.neuroimage.2022.119474 PMC9465771

[hbm70629-bib-0033] Hulvershorn, J. , L. Bloy , E. E. Gualtieri , J. S. Leigh , and M. A. Elliott . 2005. “Spatial Sensitivity and Temporal Response of Spin Echo and Gradient Echo Bold Contrast at 3 T Using Peak Hemodynamic Activation Time.” NeuroImage 24, no. 1: 216–223. 10.1016/j.neuroimage.2004.09.033. 15588613

[hbm70629-bib-0034] Hussain, S. J. , and M. V. Freedberg . 2025. “Debunking the Myth of Excitatory and Inhibitory Repetitive Transcranial Magnetic Stimulation in Cognitive Neuroscience Research.” Journal of Cognitive Neuroscience 37, no. 5: 1009–1022. 10.1162/jocn_a_02288. 39785679

[hbm70629-bib-0035] Isaacson, J. S. , and M. Scanziani . 2011. “How Inhibition Shapes Cortical Activity.” Neuron 72, no. 2: 231–243. 10.1016/j.neuron.2011.09.027. 22017986 PMC3236361

[hbm70629-bib-0036] Jenkinson, M. , C. F. Beckmann , T. E. J. Behrens , M. W. Woolrich , and S. M. Smith . 2012. “ FSL .” NeuroImage 62, no. 2: 782–790. 21979382 10.1016/j.neuroimage.2011.09.015

[hbm70629-bib-0037] Kellner, E. , B. Dhital , V. G. Kiselev , and M. Reisert . 2016. “Gibbs‐Ringing Artifact Removal Based on Local Subvoxel‐Shifts.” Magnetic Resonance in Medicine 76, no. 5: 1574–1581. 26745823 10.1002/mrm.26054

[hbm70629-bib-0038] Kim, S.‐G. , and S. Ogawa . 2012. “Biophysical and Physiological Origins of Blood Oxygenation Level‐Dependent fMRI Signals.” Journal of Cerebral Blood Flow and Metabolism: Official Journal of the International Society of Cerebral Blood Flow and Metabolism 32, no. 7: 1188–1206. 10.1038/jcbfm.2012.23. 22395207 PMC3390806

[hbm70629-bib-0039] Kukke, S. N. , R. W. Paine , C. Chao , A. C. de Campos , and M. Hallett . 2014. “Efficient and Reliable Characterization of the Corticospinal System Using Transcranial Magnetic Stimulation.” Journal of Clinical Neurophysiology 31, no. 3: 246. 10.1097/WNP.0000000000000057. 24887609 PMC4744647

[hbm70629-bib-0040] Kundu, P. , V. Voon , P. Balchandani , M. V. Lombardo , B. A. Poser , and P. A. Bandettini . 2017. “Multi‐Echo fMRI: A Review of Applications in fMRI Denoising and Analysis of BOLD Signals.” NeuroImage 154: 59–80. 28363836 10.1016/j.neuroimage.2017.03.033

[hbm70629-bib-0041] Kwon, J. , S. Lee , Y. Jo , and M. Choi . 2023. “All‐Optical Observation on Activity‐Dependent Nanoscale Dynamics of Myelinated Axons.” Neurophotonics 10, no. 1: 015003. 10.1117/1.NPh.10.1.015003. 36699624 PMC9868287

[hbm70629-bib-0042] Ling, T. , K. C. Boyle , V. Zuckerman , et al. 2020. “High‐Speed Interferometric Imaging Reveals Dynamics of Neuronal Deformation During the Action Potential.” Proceedings of the National Academy of Sciences of the United States of America 117, no. 19: 10278–10285. 10.1073/pnas.1920039117. 32341158 PMC7229674

[hbm70629-bib-0043] Logothetis, N. K. 2002. “The Neural Basis of the Blood‐Oxygen‐Level‐Dependent Functional Magnetic Resonance Imaging Signal.” Philosophical Transactions of the Royal Society, B: Biological Sciences 357, no. 1424: 1003–1037. 10.1098/rstb.2002.1114. PMC1693017 12217171

[hbm70629-bib-0044] Manjón, J. V. , P. Coupé , L. Martí‐Bonmatí , D. L. Collins , and M. Robles . 2010. “Adaptive Non‐Local Means Denoising of MR Images With Spatially Varying Noise Levels.” Journal of Magnetic Resonance Imaging 31, no. 1: 192–203. 20027588 10.1002/jmri.22003

[hbm70629-bib-0045] Mochizuki, H. , T. Furubayashi , R. Hanajima , et al. 2007. “Hemoglobin Concentration Changes in the Contralateral Hemisphere During and After Theta Burst Stimulation of the Human Sensorimotor Cortices.” Experimental Brain Research 180, no. 4: 667–675. 10.1007/s00221-007-0884-5. 17297550

[hbm70629-bib-0046] Moeller, S. , P. K. Pisharady , S. Ramanna , et al. 2021. “NOise Reduction With DIstribution Corrected (NORDIC) PCA in dMRI With Complex‐Valued Parameter‐Free Locally Low‐Rank Processing.” NeuroImage 226: 117539. 33186723 10.1016/j.neuroimage.2020.117539 PMC7881933

[hbm70629-bib-0047] Moon, H. S. , H. Jiang , T. T. Vo , W. B. Jung , A. L. Vazquez , and S. G. Kim . 2021. “Contribution of Excitatory and Inhibitory Neuronal Activity to BOLD fMRI.” Cerebral Cortex 31, no. 9: 4053–4067. 10.1093/cercor/bhab068. 33895810 PMC8328221

[hbm70629-bib-0048] Mulkern, R. V. , S. J. Haker , and S. E. Maier . 2007. “Complimentary Aspects of Diffusion Imaging and fMRI: II. Elucidating Contributions to the fMRI Signal With Diffusion Sensitization.” Magnetic Resonance Imaging 25, no. 6: 939–952. 10.1016/j.mri.2007.02.006. 17442520

[hbm70629-bib-0049] Mullinger, K. J. , S. D. Mayhew , A. P. Bagshaw , R. Bowtell , and S. T. Francis . 2014. “Evidence That the Negative BOLD Response Is Neuronal in Origin: A Simultaneous EEG–BOLD–CBF Study in Humans.” NeuroImage 94: 263–274. 10.1016/j.neuroimage.2014.02.029. 24632092

[hbm70629-bib-0050] Navarro de Lara, L. I. , C. Windischberger , A. Kuehne , et al. 2015. “A Novel Coil Array for Combined TMS/fMRI Experiments at 3 T.” Magnetic Resonance in Medicine 74, no. 5: 1492–1501. 10.1002/mrm.25535. 25421603 PMC4737243

[hbm70629-bib-0051] Newton, J. M. , A. Sunderland , and P. A. Gowland . 2005. “fMRI Signal Decreases in Ipsilateral Primary Motor Cortex During Unilateral Hand Movements Are Related to Duration and Side of Movement.” NeuroImage 24, no. 4: 1080–1087. 10.1016/j.neuroimage.2004.10.003. 15670685

[hbm70629-bib-0052] Nguyen‐Duc, J. , I. De Riedmatten , A. P. C. Spencer , J. B. Perot , W. Olszowy , and I. Jelescu . 2025. “Mapping Activity and Functional Organisation of the Motor and Visual Pathways Using ADC‐fMRI in the Human Brain.” Human Brain Mapping 46, no. 2: e70110. 10.1002/hbm.70110. 39835608 PMC11747996

[hbm70629-bib-0053] Norris, D. G. 2012. “Spin‐Echo fMRI: The Poor Relation?” NeuroImage 62, no. 2: 1109–1115. 10.1016/j.neuroimage.2012.01.003. 22245351

[hbm70629-bib-0054] Nunes, D. , R. Gil , and N. Shemesh . 2021. “A Rapid‐Onset Diffusion Functional MRI Signal Reflects Neuromorphological Coupling Dynamics.” NeuroImage 231: 117862. 33592243 10.1016/j.neuroimage.2021.117862

[hbm70629-bib-0055] Ogawa, S. , T. M. Lee , A. R. Kay , and D. W. Tank . 1990. “Brain Magnetic Resonance Imaging With Contrast Dependent on Blood Oxygenation.” Proceedings of the National Academy of Sciences of the United States of America 87, no. 24: 9868–9872. 2124706 10.1073/pnas.87.24.9868 PMC55275

[hbm70629-bib-0056] Oldfield, R. C. 1971. “The Assessment and Analysis of Handedness: The Edinburgh Inventory.” Neuropsychologia 9, no. 1: 97–113. 10.1016/0028-3932(71)90067-4. 5146491

[hbm70629-bib-0057] Peinemann, A. , B. Reimer , C. Löer , et al. 2004. “Long‐Lasting Increase in Corticospinal Excitability After 1800 Pulses of Subthreshold 5 Hz Repetitive TMS to the Primary Motor Cortex.” Clinical Neurophysiology 115, no. 7: 1519–1526. 10.1016/j.clinph.2004.02.005. 15203053

[hbm70629-bib-0058] Pérot, J.‐B. , A. Hertanu , A. Spencer , et al. 2026. “Complementarity of BOLD and ADC‐fMRI in Mapping Brain Visual Processing in the Rat.” NMR in Biomedicine 39, no. 3: e70231. 10.1002/nbm.70231. 41631514 PMC12865745

[hbm70629-bib-0059] Philip, N. S. , S. L. Carpenter , S. J. Ridout , et al. 2015. “5 Hz Repetitive Transcranial Magnetic Stimulation to Left Prefrontal Cortex for Major Depression.” Journal of Affective Disorders 186: 13–17. 10.1016/j.jad.2014.12.024. 26210705 PMC4565741

[hbm70629-bib-0060] Philip, N. S. , S. J. Ridout , S. E. Albright , G. Sanchez , and L. L. Carpenter . 2016. “5‐Hz Transcranial Magnetic Stimulation for Comorbid Posttraumatic Stress Disorder and Major Depression.” Journal of Traumatic Stress 29, no. 1: 93–96. 10.1002/jts.22065. 26748883 PMC4849266

[hbm70629-bib-0061] Power, J. D. , K. A. Barnes , A. Z. Snyder , B. L. Schlaggar , and S. E. Petersen . 2012. “Spurious but Systematic Correlations in Functional Connectivity MRI Networks Arise From Subject Motion.” NeuroImage 59, no. 3: 2142–2154. 22019881 10.1016/j.neuroimage.2011.10.018 PMC3254728

[hbm70629-bib-0062] Quartarone, A. , S. Bagnato , V. Rizzo , et al. 2005. “Distinct Changes in Cortical and Spinal Excitability Following Highfrequency Repetitive TMS to the Human Motor Cortex.” Experimental Brain Research 161, no. 1: 114–124. 10.1007/s00221-004-2052-5. 15578171

[hbm70629-bib-0063] Rossi, S. , M. Hallett , P. M. Rossini , et al. 2009. “Safety, Ethical Considerations, and Application Guidelines for the Use of Transcranial Magnetic Stimulation in Clinical Practice and Research.” Clinical Neurophysiology 120, no. 12: 2008–2039. 10.1016/j.clinph.2009.08.016. 19833552 PMC3260536

[hbm70629-bib-0064] Rossini, P. M. , D. Burke , R. Chen , et al. 2015. “Non‐Invasive Electrical and Magnetic Stimulation of the Brain, Spinal Cord, Roots and Peripheral Nerves: Basic Principles and Procedures for Routine Clinical and Research Application. An Updated Report From an I.F.C.N. Committee.” Clinical Neurophysiology 126, no. 6: 1071–1107. 10.1016/j.clinph.2015.02.001. 25797650 PMC6350257

[hbm70629-bib-0065] Saitoh, Y. , A. Hirayama , H. Kishima , et al. 2007. “Reduction of Intractable Deafferentation Pain due to Spinal Cord or Peripheral Lesion by High‐Frequency Repetitive Transcranial Magnetic Stimulation of the Primary Motor Cortex.” Journal of Neurosurgery 107, no. 3: 555–559. 10.3171/JNS-07/09/0555. 17886555

[hbm70629-bib-0066] Schridde, U. , M. Khubchandani , J. E. Motelow , B. G. Sanganahalli , F. Hyder , and H. Blumenfeld . 2008. “Negative BOLD With Large Increases in Neuronal Activity.” Cerebral Cortex 18, no. 8: 1814–1827. 10.1093/cercor/bhm208. 18063563 PMC2790390

[hbm70629-bib-0067] Sherpa, A. D. , F. Xiao , N. Joseph , C. Aoki , and S. Hrabetova . 2016. “Activation of β‐Adrenergic Receptors in Rat Visual Cortex Expands Astrocytic Processes and Reduces Extracellular Space Volume.” Synapse 70, no. 8: 307–316. 10.1002/syn.21908. 27085090 PMC4909535

[hbm70629-bib-0068] Shimomura, T. , M. Fujiki , H. Ohba , et al. 2018. “Contralateral Negative Bold Responses in the Motor Network During Subthreshold High‐Frequency Interleaved TMS‐fMRI Over the Human Primary Motor Cortex.” Journal of Neurology & Neurophysiology 9, no. 6: 1–8. 10.4172/2155-9562.1000478.

[hbm70629-bib-0069] Shmuel, A. , M. Augath , A. Oeltermann , and N. K. Logothetis . 2006. “Negative Functional MRI Response Correlates With Decreases in Neuronal Activity in Monkey Visual Area V1.” Nature Neuroscience 9, no. 4: 569–577. 10.1038/nn1675. 16547508

[hbm70629-bib-0072] Siebner, H. R. , K. Funke , A. S. Aberra , et al. 2022. “Transcranial Magnetic Stimulation of the Brain: What Is Stimulated?—A Consensus and Critical Position Paper.” Clinical Neurophysiology 140: 59–97. 10.1016/j.clinph.2022.04.022. 35738037 PMC9753778

[hbm70629-bib-0070] Siebner, H. R. , C. Mentschel , C. Auer , and B. Conrad . 1999. “Repetitive Transcranial Magnetic Stimulation Has a Beneficial Effect on Bradykinesia in Parkinson's Disease.” Neuroreport 10, no. 3: 589–594. 10.1097/00001756-199902250-00027. 10208595

[hbm70629-bib-0071] Siebner, H. R. , C. Mentschel , C. Auer , C. Lehner , and B. Conrad . 2000. “Repetitive Transcranial Magnetic Stimulation Causes a Short‐Term Increase in the Duration of the Cortical Silent Period in Patients With Parkinson's Disease.” Neuroscience Letters 284, no. 3: 147–150. 10.1016/s0304-3940(00)00990-3. 10773420

[hbm70629-bib-0073] Siebner, H. R. , TO Bergmann , S. Bestmann , et al. 2009. “Consensus Paper: Combining Transcranial Stimulation With Neuroimaging.” Brain Stimulation 2, no. 2: 58–80. 10.1016/j.brs.2008.11.002. 20633405

[hbm70629-bib-0074] Spees, W. M. , T.‐H. Lin , and S.‐K. Song . 2013. “White‐Matter Diffusion fMRI of Mouse Optic Nerve.” NeuroImage 65: 209–215. 23085108 10.1016/j.neuroimage.2012.10.021 PMC3513522

[hbm70629-bib-0075] Spencer, A. P. C. , J. Nguyen‐Duc , I. de Riedmatten , F. Szczepankiewicz , and I. O. Jelescu . 2025. “Mapping Grey and White Matter Activity in the Human Brain With Isotropic ADC‐fMRI.” Nature Communications 16, no. 1: 5036. 10.1038/s41467-025-60357-5. PMC12125227 40447634

[hbm70629-bib-0076] Stefanovic, B. , J. M. Warnking , and G. B. Pike . 2004. “Hemodynamic and Metabolic Responses to Neuronal Inhibition.” NeuroImage 22, no. 2: 771–778. 10.1016/j.neuroimage.2004.01.036. 15193606

[hbm70629-bib-0077] Su, T.‐P. , C.‐C. Huang , and I.‐H. Wei . 2005. “Add‐On rTMS for Medication‐Resistant Depression: A Randomized, Double‐Blind, Sham‐Controlled Trial in Chinese Patients.” Journal of Clinical Psychiatry 66, no. 7: 930–937. 10.4088/jcp.v66n0718. 16013911

[hbm70629-bib-0078] Sundqvist, N. , H. Podéus , S. Sten , et al. 2025. “Model‐Driven Meta‐Analysis Establishes a New Consensus View: Inhibitory Neurons Dominate BOLD‐fMRI Responses.” Computers in Biology and Medicine 197: 111014. 10.1016/j.compbiomed.2025.111014. 40926439

[hbm70629-bib-0079] Szczepankiewicz, F. , J. Sjölund , F. Ståhlberg , J. Lätt , and M. Nilsson . 2019. “Tensor‐Valued Diffusion Encoding for Diffusional Variance Decomposition (DIVIDE): Technical Feasibility in Clinical MRI Systems.” PLoS One 14, no. 3: e0214238. 10.1371/journal.pone.0214238. 30921381 PMC6438503

[hbm70629-bib-0080] Szczepankiewicz, F. , C.‐F. Westin , and M. Nilsson . 2019. “Maxwell‐Compensated Design of Asymmetric Gradient Waveforms for Tensor‐Valued Diffusion Encoding.” Magnetic Resonance in Medicine 82, no. 4: 1424–1437. 10.1002/mrm.27828. 31148245 PMC6626569

[hbm70629-bib-0081] Takeuchi, N. , Y. Oouchida , and S.‐I. Izumi . 2012. “Motor Control and Neural Plasticity Through Interhemispheric Interactions.” Neural Plasticity 2012: 823285. 10.1155/2012/823285. 23326685 PMC3541646

[hbm70629-bib-0082] Tournier, J.‐D. , R. Smith , D. Raffelt , et al. 2019. “MRtrix3: A Fast, Flexible and Open Software Framework for Medical Image Processing and Visualisation.” NeuroImage 202: 116137. 31473352 10.1016/j.neuroimage.2019.116137

[hbm70629-bib-0083] Tustison, N. J. , B. B. Avants , P. A. Cook , et al. 2010. “N4ITK: Improved N3 Bias Correction.” IEEE Transactions on Medical Imaging 29, no. 6: 1310–1320. 10.1109/TMI.2010.2046908. 20378467 PMC3071855

[hbm70629-bib-0084] Tustison, N. J. , P. A. Cook , A. J. Holbrook , et al. 2021. “The ANTsX Ecosystem for Quantitative Biological and Medical Imaging.” Scientific Reports 11, no. 1: 9068. 33907199 10.1038/s41598-021-87564-6 PMC8079440

[hbm70629-bib-0085] Virtanen, P. , R. Gommers , T. E. Oliphant , et al. 2020. “SciPy 1.0: Fundamental Algorithms for Scientific Computing in Python.” Nature Methods 17, no. 3: 261–272. 10.1038/s41592-019-0686-2. 32015543 PMC7056644

[hbm70629-bib-0086] Wade, A. R. 2002. “The Negative BOLD Signal Unmasked.” Neuron 36, no. 6: 993–995. 10.1016/s0896-6273(02)01138-8. 12495615

[hbm70629-bib-0087] Wang, J. , Y. Yang , L. Fan , et al. 2014. “Convergent Functional Architecture of the Superior Parietal Lobule Unraveled With Multimodal Neuroimaging Approaches.” Human Brain Mapping 36, no. 1: 238–257. 10.1002/hbm.22626. 25181023 PMC4268275

[hbm70629-bib-0090] Woolrich, M. W. , B. D. Ripley , M. Brady , and S. M. Smith . 2001. “Temporal Autocorrelation in Univariate Linear Modeling of FMRI Data.” NeuroImage 14, no. 6: 1370–1386. 10.1006/nimg.2001.0931. 11707093

[hbm70629-bib-0091] Woolrich, M. W. , T. E. J. Behrens , C. F. Beckmann , M. Jenkinson , and S. M. Smith . 2004. “Multilevel Linear Modelling for FMRI Group Analysis Using Bayesian Inference.” NeuroImage 21, no. 4: 1732–1747. 10.1016/j.neuroimage.2003.12.023. 15050594

[hbm70629-bib-0088] Zhou, Q. , K. J. Homma , and M.‐m. Poo . 2004. “Shrinkage of Dendritic Spines Associated With Long‐Term Depression of Hippocampal Synapses.” Neuron 44, no. 5: 749–757. 10.1016/j.neuron.2004.11.011. 15572107

